# Computational-experimental study reveals direct target and bioactives of *Ajania fruticulosa* against NAFLD via TLR2/NF-κB/PPAR-γ signaling

**DOI:** 10.1038/s41538-026-00722-w

**Published:** 2026-01-23

**Authors:** Chaoyue Chen, Lisha Ma, Awaguli Dawuti, Xin Feng, Shujie Chen, Xueyan An, Yulan Bai, Tianfeng Zhang, Mamatjan Aydin, Kashif Kashmiri, Zhancang Ma, Wei Zhang, Saimijiang Yaermaimaiti, Abudumijiti Abulizi

**Affiliations:** 1 Pharmacy College of Shihezi University/Key Laboratory of Xinjiang Phytomedicine Resource and Utilization, Ministry of Education/Collaborative Innovation Center for Efficient Safflower Production and Resource Utilization of XPCC/Institute for Safflower Industry Research, Shihezi University, Shihezi, China; 2 Uyghur Medicines Hospital of Xinjiang Uyghur Autonomous Region, Urumqi, China; 3 Key Laboratory of Xinjiang Phytomedicine Resource and Utilization of Ministry of Education, College of Life Sciences, Shihezi University, Shihezi, China

**Keywords:** Computational biology and bioinformatics, Diseases, Drug discovery

## Abstract

Non-alcoholic fatty liver disease (NAFLD) is a prevalent metabolic disorder with limited treatment options. This study investigated the therapeutic potential of water extract of *Ajania fruticulosa* (WEAF) against NAFLD in cellular and animal models. WEAF significantly attenuated obesity, lipid accumulation, liver injury, and inflammation in NAFLD mice. Next, UPLC-MS/MS-based network pharmacology and molecular biology revealed that WEAF alleviated NAFLD by TLR2-mediated MYD88/NF-κB and SREBP1/PPAR-γ pathways, with 3,4-dihydroxyphenylpropionic acid, glycitein, and isorhapontigenin identified as the primary bioactive compounds. Finally, molecular docking, molecular dynamics, drug affinity responsive target stability, and cellular thermal shift assay confirmed that glycitein and isorhapontigenin directly bind to TLR2 to modulate the NF-κB/PPAR-γ signaling, and their anti-NAFLD effects were abolished by TLR2 agonist Pam3CSK4. In conclusion, WEAF and its key active compounds, glycitein and isorhapontigenin, effectively ameliorate obesity-induced NAFLD via the NF-κB/PPAR-γ signaling pathway by targeting TLR2, supporting their potential as therapeutic target and agents for NAFLD.

## Introduction

Non-alcoholic fatty liver disease (NAFLD) represents a pathological situation where lipids accumulate mainly in hepatocytes. This condition is linked to non-inflammatory steatosis and lobular inflammatory alterations. It has emerged as the most common liver disorder globally. Prevalence estimates show that it affects 31.6% of the population in Asia, 32.6% in Europe, 47.8% in North America, and 56.8% in Africa^1,2^. NAFLD can develop into liver cirrhosis and liver cancer if there is no effective treatment^3^. Currently, Rezdiffra (resmetirom) and semaglutide (Wegovy) have received accelerated FDA approval for this indication^4,5^. However, their associated adverse effects, including hepatotoxicity and gallbladder or gastrointestinal disorders, underscore the need for more effective and less toxic therapeutic agents.

Currently, the etiopathogenesis of NAFLD is not fully elucidated and is relatively complicated, including inflammation, lipid metabolism disorder, insulin resistance, endoplasmic reticulum stress, mitochondrial dysfunction, oxidative stress, gut microbiota, and genetics^6^. It has been found that inflammation and hepatic lipid disorder are the key factors in the development of NAFLD^7,8^. TLR-mediated intrinsic immune response has been activated in the progression of NASH^9^, and its over-activation produces excessive amounts of inflammatory cytokines through regulating NF-κB signaling^10^. NF-κB is activated in damaged hepatocytes and induces the expression of pro-inflammatory cytokines such as TNF-α, IL-6, and IL-1β, which further exacerbate the inflammatory response and hepatocyte injury^11,12^. Hepatic lipid accumulation is subject to adipose sterol regulatory element binding protein 1c (SREBP1c), which promotes the expression of downstream target genes, fatty acid synthetase (FAS), and acetyl coenzyme A carboxylase (ACC)^13,14^. PPAR-γ is a core nuclear receptor for glucose and lipid homeostasis^15^, and is up-regulated in the livers of obese NAFLD patients, and enhanced lipogenesis induced by SREBP-1c in hepatic steatosis development^16^.

For several decades, traditional Chinese medicine (TCM) has been widely utilized to treat different metabolic disorders, including NAFLD and anti-inflammatory disease^17^. *Ajania fruticulosa* (AF) is abundant in northwestern China and has properties that clear heat and detoxify, promote blood circulation and remove blood stasis, relieve coughs, soothe the throat, relieve pain, and reduce inflammation^18^. Pharmacological studies have reported that it has anti-inflammatory, antioxidant, anticancer, and bacteriostatic effects^19,20^. It has been reported that flavonoids, triterpenes, sterols, etc., are the main chemical components of AF, including 7-desmethylartemetin, ketopelenolide B, artecanin, pectolinarigenin, cirsiliol, etc.^21^. Glycitein significantly altered NF-κB and MAPK pathway activities and significantly reduced the phosphorylation levels of NF-κB subunits p65 and p50, whereas isorhapontigenin significantly attenuated acetaminophen-induced hepatic injury by effectively inhibiting apoptosis, oxidative stress, and inflammation^22,23^. The above reports indicated that AF may have a potential effect against liver disease, but its role and mechanism have not been elucidated.

In this study, we investigated the effects of water extract of *Ajania fruticulosa* (WEAF) on high-fat diet-induced NAFLD and its intrinsic mechanisms through network pharmacology and experimental validation. First, we investigated the anti-NAFLD effects of WEAF in FFA-stimulated HepG2 cells and obese mice. Then, the main components of WEAF were identified using UPLC-MS/MS. Subsequently, the pivotal targets and related pathways of WEAF against NAFLD were predicted by systems pharmacology approach leveraging multiple databases. The predictions were corroborated by molecular docking and molecular biology experiments, revealing that WEAF attenuates NAFLD through the TLR2/NF-κB/PPAR-γ signaling pathway, with 3,4-Dihydroxyphenylpropionic acid, Glycitein, and Isorhapontigenin being the major active compounds of WEAF. Besides, molecular docking, molecular dynamics, cellular thermal shift assay (CETSA), and drug affinity responsive target stability (DARTS) validation confirmed that Glycitein and Isorhapontigenin could exert anti-NAFLD effects by targeting TLR2. Finally, the TLR2 agonist Pam3CSK4 abolished their anti-NAFLD effects.

## Results

### WEAF significantly relieved intracellular TG and TC content and lipid accumulation

**Fig. 1:**
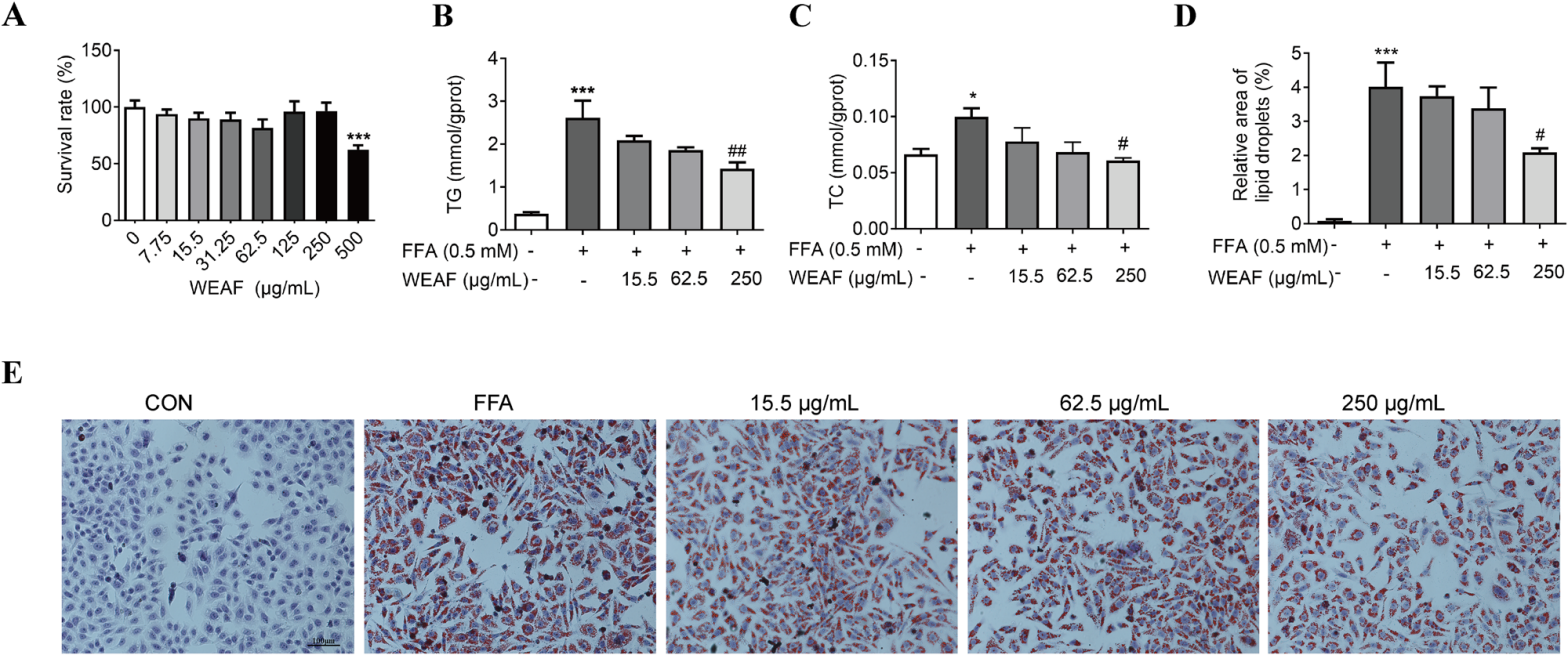
WEAF significantly reduced intracellular TG and TC content and lipid accumulation. **A** Cell survival rate. **B** TG content. **C** TC content. **D** Relative area of lipid droplets. **E** Oil red O staining of HepG2 cells (scale bar: 100 μm ×20 magnification). Mean ± SEM, *n* = 3,*p < 0.05, ***p < 0.001 vs. control group; ^#^*p* < 0.05, ^#^^#^*p* < 0.01 vs. FFA group. L-D Low dose, M-D medium dose, H-D high dose, ATO Atorvastatin.

To test the effect of WEAF on cell viability, we performed a CCK-8 assay and found that WEAF had no significant effect on cell viability at concentrations up to 250 μg/mL (Fig. 1A). Next, we studied if WEAF had a potential effect in in vitro NAFLD model, and found that WEAF significantly reduced intracellular TG and TC content in FFA-stimulated HepG2 cells in a dose-dependent manner compared to the FFA-stimulated model group (Fig. 1B, C). In addition, the results of Oil red O staining showed that WEAF significantly reduced FFA-induced intracellular lipid accumulation at a concentration of 250 μg/mL compared with the FFA-treated model group (Fig. 1D, E). The above results suggest that WEAF may have a potential role in NAFLD.

### WEAF attenuates HFD-induced obesity and liver function in mice

**Fig. 2:**
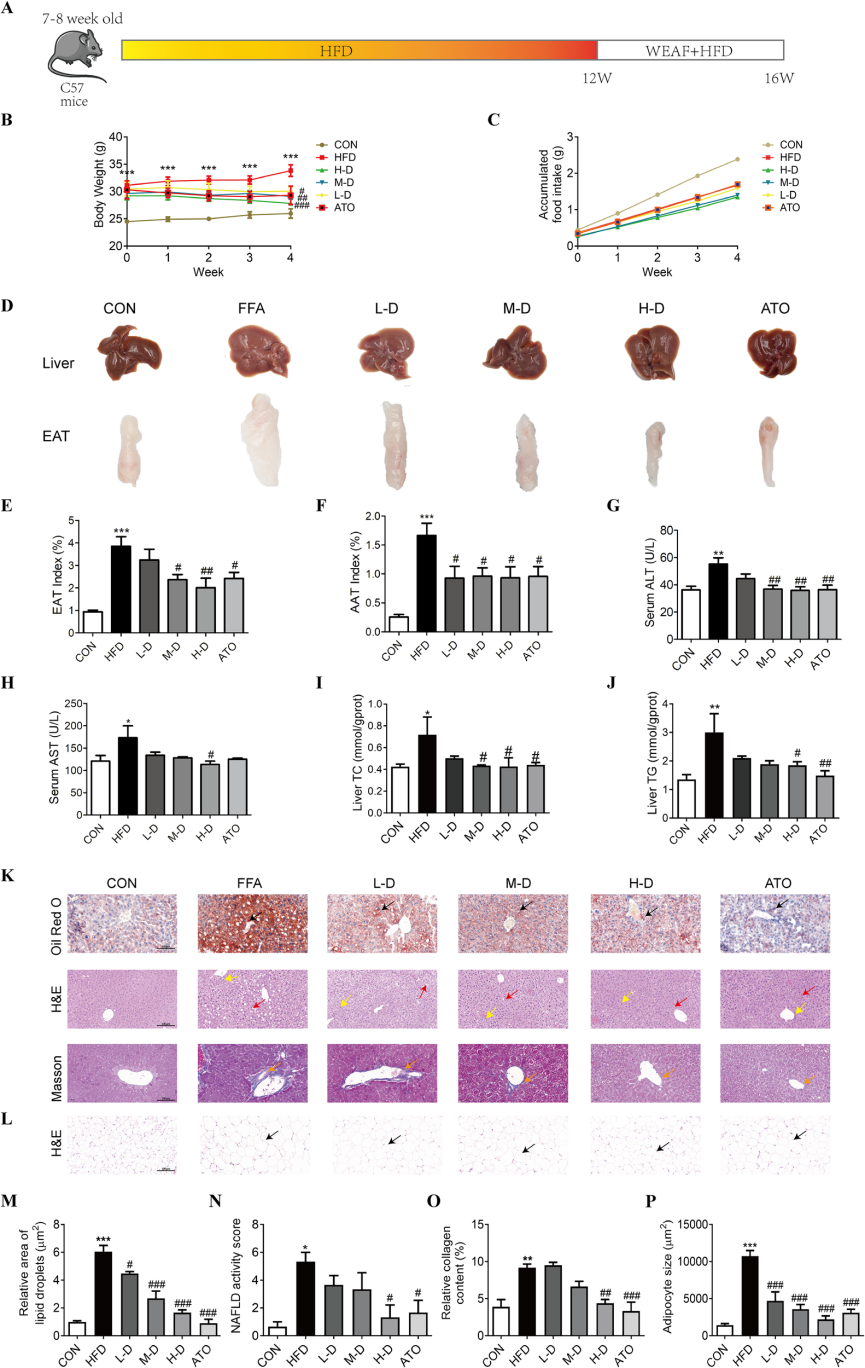
WEAF ameliorates high fat diet–induced obesity, liver dysfunction, and histopathological injuries in NAFLD mice. **A** Experimental flow of HFD-induced NAFLD mouse model and WEAF treatment. **B** Body weight. **C** Food intake. **D** Macromorphological observation of liver and epididymal fat. **E** Epididymal fat index. **F** Abdominal fat index. **G** Serum ALT level. **H** Serum AST level. **I** TC content in the liver. **J** TG content in the liver. **K** Oil Red O staining, H&E staining, and Masson staining of liver tissue (Scale bar: 100 μm ×20 magnification) (Black arrow: Oil-red-stained lipid droplets; Red arrow: Lipid droplet vacuoles; Yellow arrow: Inflammatory infiltration; Orange arrow: Collagen fibers). **L** H&E staining of epididymal fat tissue (Black arrow: Enlarged adipocyte). **M** Relative area of lipid droplets. **N** NAFLD activity score. **O** Relative collagen content. **P** adipocytes size. Mean ± SEM, *n* = 10 for panels (**B**–**J**), *n* = 3 for panels (**K**–**P**); *p < 0.05, **p < 0.001, ***p < 0.001 vs. CON group; ^#^*p* < 0.05; ^##^*p* < 0.01; ^###^*p* < 0.01 vs. HFD group. L-D Low dose, M-D medium dose, H-D high dose, ATO Atorvastatin.

To establish an HFD-induced NAFLD model, mice were given a high-fat diet (HFD) for 12 weeks, and WEAF and ATO were administered for an additional 4 weeks (Fig. 2A). The results revealed that the mice in the HFD group weighed more than the mice in the control group, and the body weight of the HFD mice group treated with WEAF and ATO significantly reduced in a dose-dependent manner (Fig. 2B). The accumulative food intake of HFD mice did not significantly change after WEAF treatment (Fig. 2C). The color of liver in the HFD group became whiter than the control group, with more obvious granulation and rougher surface, while WEAF and ATO administration significantly improved the above changes, including mouse livers became red and smooth surface (Fig. 2D). We also found that the epididymis and abdominal fat index were significantly increased in the HFD group, and these increases were notably attenuated in the WEAF and ATO groups (Fig. 2E, F). Levels of serum ALT, AST, and liver TC and TG significantly increased in the HFD group, while WEAF treatment decreased serum levels of ALT, AST, and liver TG and TC content in HFD mice (Fig. 2G–J).

### WEAF attenuates histopathological injury of liver and adipose tissue in NAFLD mice

Next, we detected the effect of WEAF against NAFLD-induced histopathological injury, and the results of liver Oil red O staining showed that HFD mice showed excessive red lipid droplets in the liver, which were significantly ameliorated after WEAF and ATO administration in a dose-dependent manner (Fig. 2K, M). In addition, H&E staining of the HFD group revealed a large number of liver lipid vacuoles and inflammatory cell infiltration, which was significantly attenuated after WEAF treatment in HFD mice (Fig. 2K, N). Moreover, Masson staining demonstrated that there was evident collagen deposition around the liver central vein in the HFD group, and the collagen deposition was reduced after the administration of WEAF and ATO (Fig. 2K, O). Then, H&E staining of epididymal fat showed that adipocytes in the control group were tightly arranged, uniform in size, and small, while adipocytes in the HFD group were hypertrophied with fuzzy edges and irregular shapes, and the above abnormal changes were strikingly improved after WEAF and ATO treatment (Fig. 2L, P). These results show that WEAF significantly attenuates HFD-induced obesity and NAFLD, including liver steatosis, steatohepatitis, and fibrosis.

### Chemical components of WEAF

**Fig. 3:**
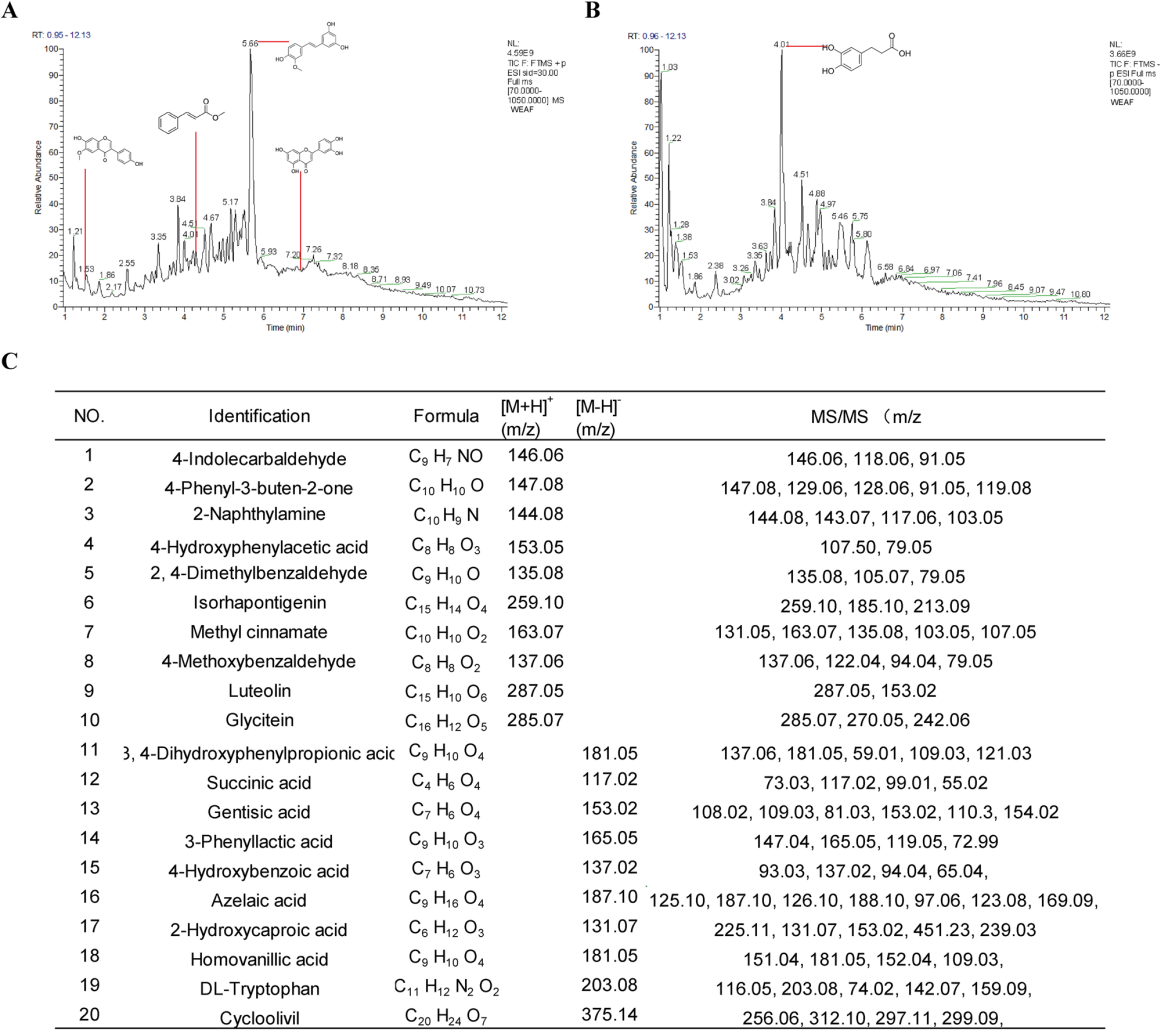
Chemical components of WEAF. **A** Positive ion mode. **B** Negative ion mode. **C** Information on compounds identified by UPLC/MS/MS.

A total of 20 compounds were identified as the main components of WEAF by using UPLC-MS/MS through mass fragmentation patterns in comparison to the literature (Fig. 3A, B), which including 4-Indolecarbaldehyde, 4-Phenyl-3-buten-2-one, 2-Naphthylamine, 4-Hydroxyphenylacetic acid, 2,4-Dimethylbenzaldehyde, Isorhapontigenin, Methyl cinnamate, 4-Methoxybenzaldehyde, Luteolin, Glycitein, 3,4-Dihydroxyphenylpropionic acid, Succinic acid, Gentisic acid, 3-Phenyllactic acid, 4-Hydroxybenzoic acid, Azelaic acid, 2-Hydroxycaproic acid, Homovanillic acid, DL-Tryptophan, Cycloolivil (Fig. 3C), indicating those may be the material bases of WEAF against NAFLD.

### Results of network pharmacology analyses

**Fig. 4:**
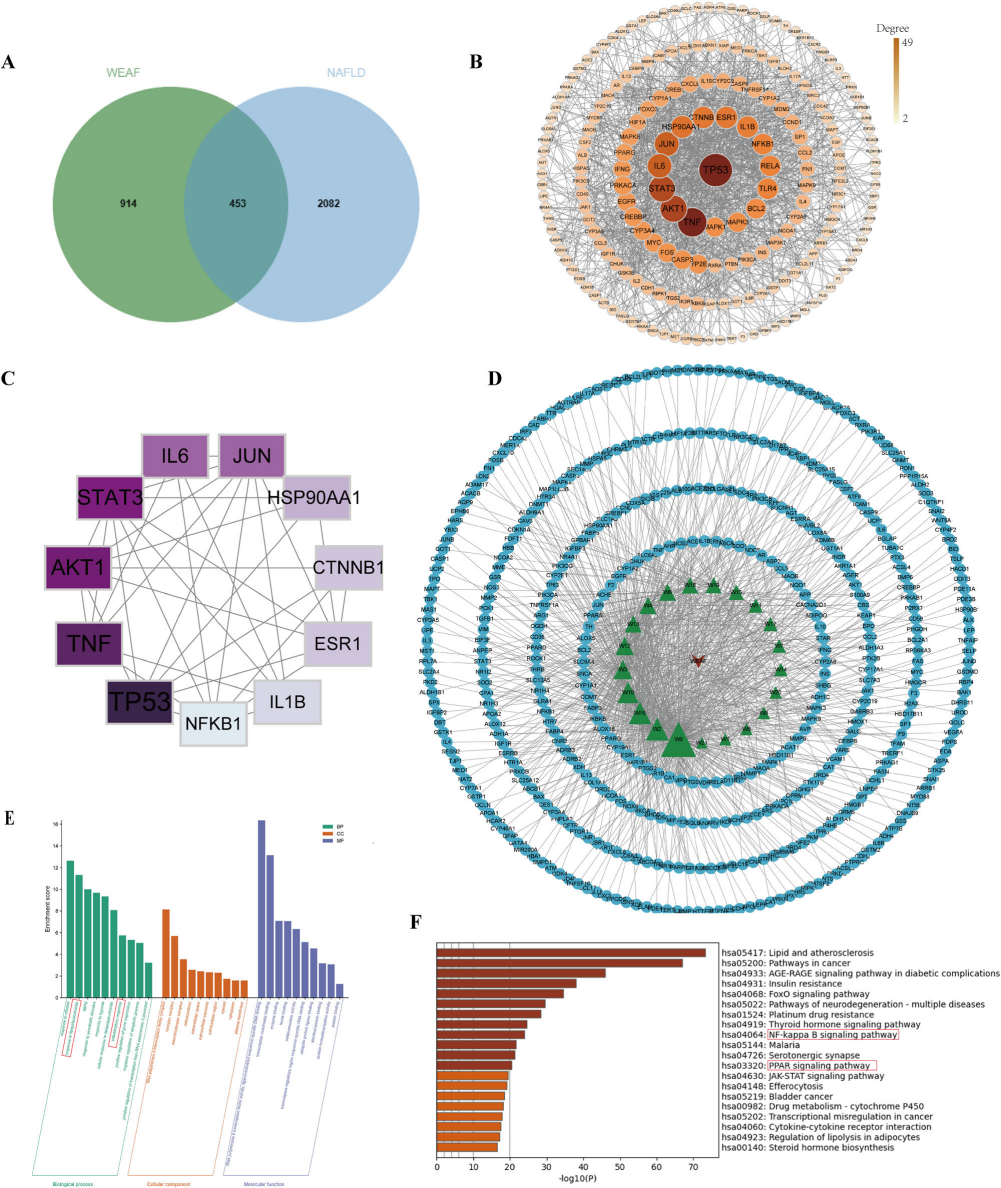
Results of network pharmacological analyses. **A** Venn Diagram of WEAF and NAFLD. **B** PPI network. **C** The top hub genes. **D** Drug-component-target network diagram. **E** GO enrichment analysis. **F** KEGG analysis.

The identified main compounds from WEAF by UPLC/MS/MS were used to study network pharmacology. We obtained a total of 1396 potential targets for WEAF and 2534 potential targets for NAFLD based on related databases mentioned in Methods, and 453 overlap genes were found between WEAF and NAFLD (Fig. 4A), suggesting that 453 genes could be the key targets of WEAF against NAFLD. A “target-target network” was created using STRING data from 453 overlapping genes with high confidence (>0.7). The PPI network comprises 475 nodes and 959 edges, as shown in Fig. 4B, Node color represents degree values, with a gradient from dark to light indicating high to low degrees. Node size corresponds to degree values, where larger sizes represent higher degrees. Edges represent protein-protein interactions. The top 10 hub genes included *TP53, TNF, IL-6, AKT1, IL-1β, STAT3, EGFR, INS, CASP3*, and *NFκB1*, which are likely to be key targets of WEAF against NAFLD (Fig. 4C). To further identify which components mainly play the anti-NAFLD effect, 20 compound were subjected to PPI network interactions with the core targets, and the results showed that W9 (Luteolin), W2 (4-Phenyl-3-buten-2-one), W16 (Azelaic acid) W10 (Glycitein), W3 (2-Naphthylamine), W12 (Succinic acid), W13 (Gentisic acid), W4 (4-Hydroxyphenylacetic acid), W6 (Isorhapontigenin), W18 (Homovanillic acid) may be the main active compound of WEAF. In the drug component-target network, triangles represent bioactive compounds from WEAF, and ellipses represent potential targets. Nodes are colored by type (compound or target), and edges indicate predicted compound-target interactions (Fig. 4D). Besides, the results of GO enrichment analysis showed that the mechanism of WEAF against NAFLD is mainly related to the regulation of inflammatory response, response to lipopolysaccharide, etc. (Fig. 4E). The findings of KEGG pathway analysis demonstrated that the top pathway involves AGE-RAGE, FoxO, NF-κB, PPAR, JAK-STAT3, etc. (Fig. 4F). These results suggest that WEAF may attenuate NAFLD through regulating inflammatory response and lipid metabolism via multiple pathways, including NF-κB and PPAR signaling.

### WEAF inhibits NAFLD-induced liver inflammation via TLR2/MYD88/NF-κB signaling pathway

**Fig. 5:**
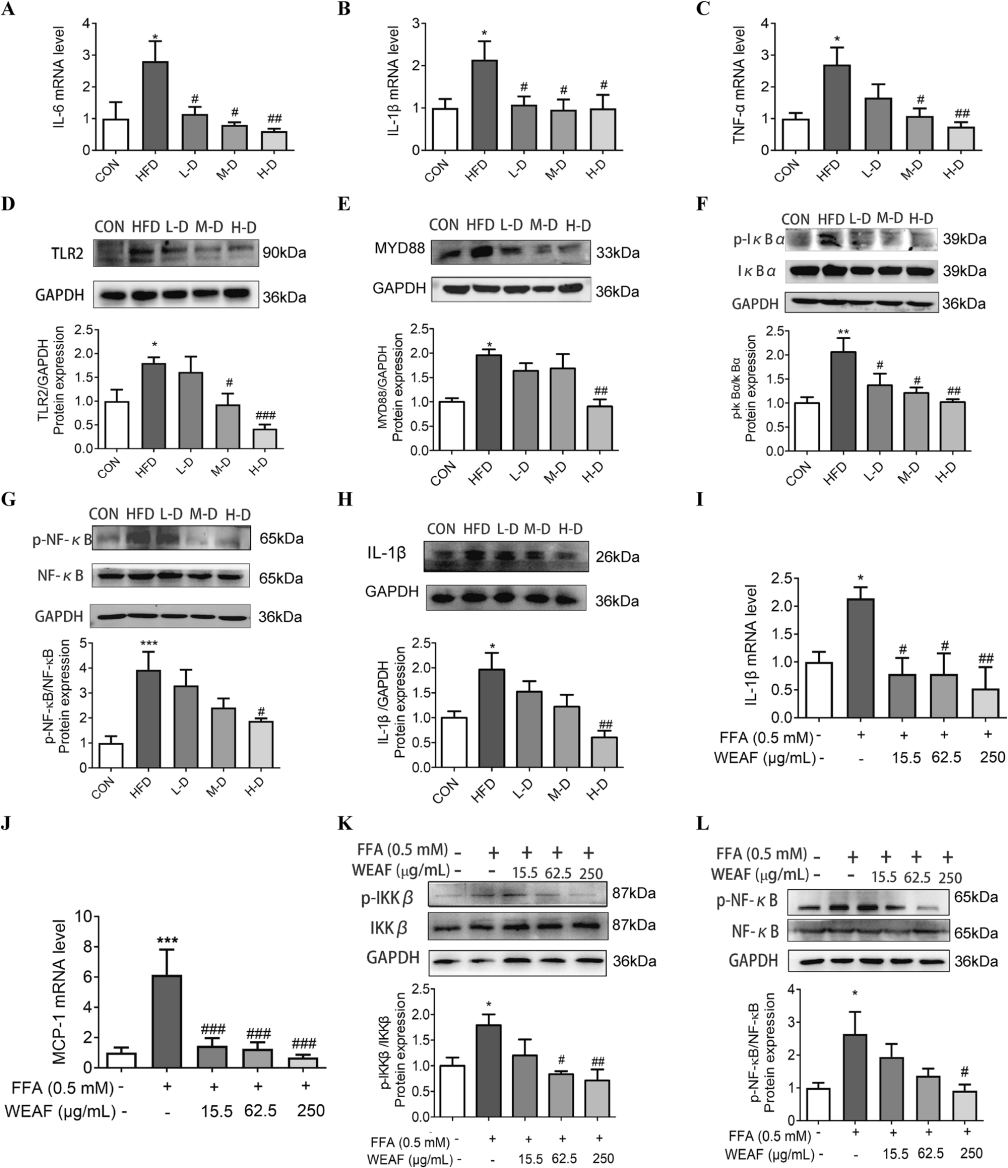
WEAF inhibits steatohepatitis via the TLR2/MYD88/NF-κB signaling pathway. **A**–**C** mRNA expression of IL-6, IL-1β and TNF-α in liver. **D**–**H** Protein expression of TLR2, MYD88, p-IκBα, IκBα, MYD88, p-NF-κB, NF-κB and IL-1β. **I**, **J** mRNA expression of IL-1β, MCP-1. **K**, **L** Protein expression of p-IKKβ, IKKβ, p-NF-κB, and NF-κB. Mean ± SEM, *n* = 6 for RT-qPCR; *n* = 4 or 5 for Western blotting; ***p < 0.001, **p < 0.01, *p < 0.05 vs. CON group. ^###^*p* < 0.001, ^##^*p* < 0.01, ^#^*p* < 0.05 vs. FFA group. L-D Low dose, M-D medium dose, H-D high dose.

GO enrichment and PPI network analysis predicted that the effect of WEAF against NAFLD may be related to inflammation response with cytokines IL-6, TNF-α, and IL-1β. Therefore, we first detected the mRNA expression of IL-6, TNF-α, and IL-1β in liver tissue, and found that WEAF significantly downregulated the mRNA level of IL-6, IL-1β, and TNF-α in the liver of HFD mice (Fig. 5A–C). Studies reported a close relationship between the NF-κB signaling pathway and cytokine release, and network pharmacological results also showed that NF-κB is one of the important pathways. Therefore, next, we investigated NF-κB pathway-related proteins, and found that WEAF, indeed, downregulated the protein level of TLR2, MYD88, p-IκBα, p-NF-κB, and IL-1β in the liver of HFD mice (Fig. 5D–H). Moreover, we also studied whether WEAF inhibits the inflammation in FFA-stimulated HepG2 cells, and found that WEAF not only significantly inhibited the mRNA level of IL-β and MCP-1, but also decreased the protein expression of p-IKKβ and p-NF-κB in FFA-stimulated HepG2 cells (Fig. 5I–L). The above results suggest that WEAF, at least in part, retards NAFLD through regulating inflammation response via TLR2/MYD88/NF-κB signaling pathway.

### WEAF attenuates NAFLD via SREBP1/PPAR-γ signaling pathway

**Fig. 6:**
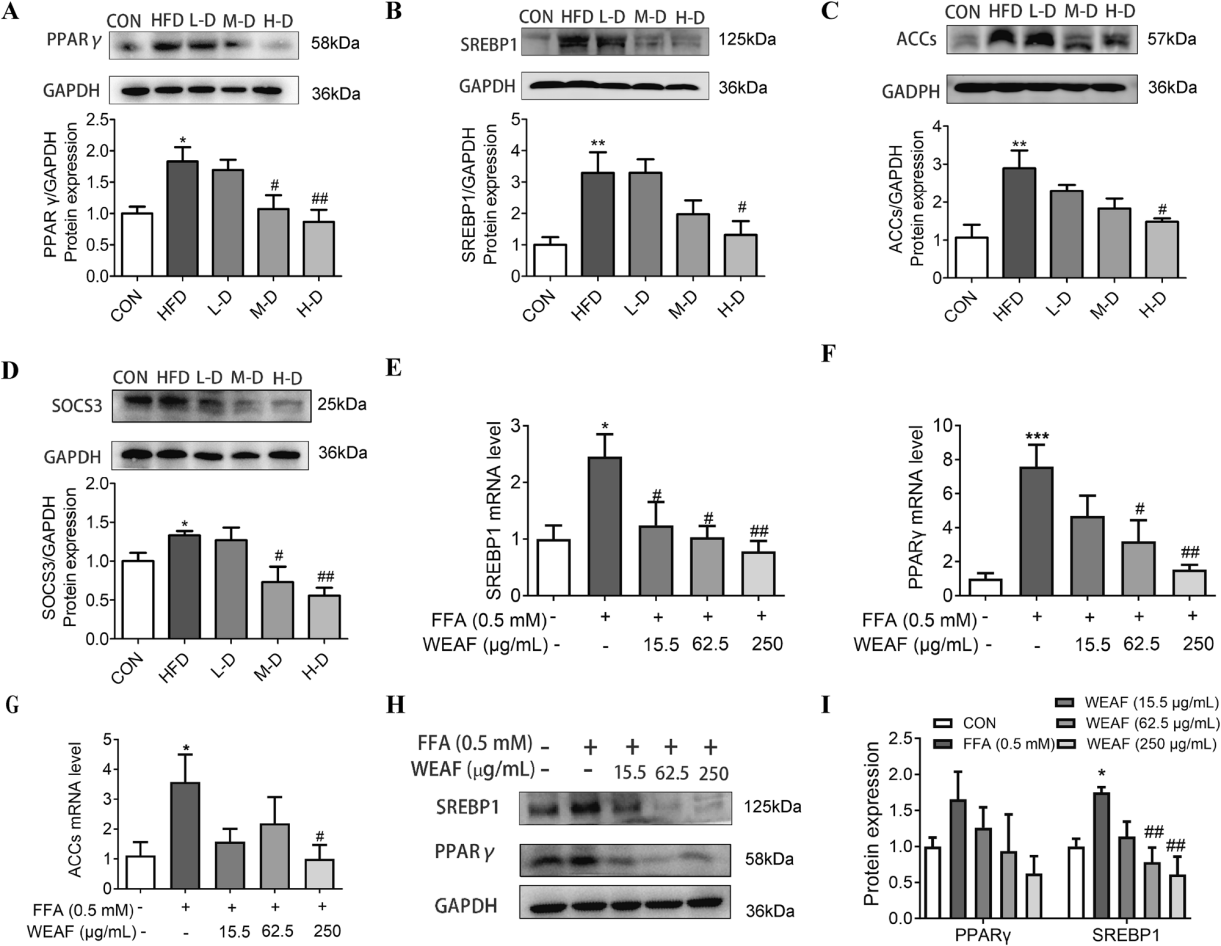
WEAF attenuates NAFLD via the SREBP1/PPAR signaling pathway. **A**–**D** Protein expression level of PPARγ, SREBP1, ACCs, and SOCS3 in liver. **E**–**G** mRNA expression of SREBP1, PPARγ and ACCs in FFA-stimulated HeG2 cell. **H**, **I** Protein expression of PPARγ and SREBP1 in FFA-stimulated HeG2 cells. Mean ± SEM, *n* = 6 for RT-qPCR; *n* = 4 or 5 for Western blotting; ***p < 0.001, **p < 0.01, *p < 0.05 vs. CON group. ^##^*p* < 0.01, ^#^*p* < 0.05 vs. FFA grou*p*. L-D Low dose, M-D medium dose, H-D high dose, ATO Atorvastatin.

It is well known that lipid metabolic disorder is one of the main pathophysiology of NAFLD, and network pharmacology results also revealed that the effect of WEAF against NAFLD may be related to the PPAR signaling pathway. Therefore, we further detected PPAR-related lipid synthesis factor PPAR-γ, SREBP1, and ACCs in vivo and in vitro, and found that the protein expression of PPAR-γ, SREBP1, ACCs, and SOCS3 in the liver of HFD mice was increased when compared to the control group, while WEAF treatment significantly inhibited the expression of those lipid synthesis-related proteins (Fig. 6A–D). Moreover, the mRNA level of PPAR-γ, SREBP1, ACCs (Fig. 6E–G), and protein expression of SREBP1 (Fig. 6H, I) were also significantly increased in FFA-simulated HePG2 cells, but WEAF treatment obviously inhibited the abnormal expression of those genes or proteins in FFA-stimulated HepG2 cells, suggesting that WEAF attenuates hepatocyte metabolic disorder via the SREBP1/PPAR signaling pathway.

### Binding stability of the main compounds of WEAF with hub genes

Network pharmacology identified 3,4-dihydroxyphenylpropionic acid, luteolin, methyl cinnamate, glycitein, and isorhapontigenin as potential active components of WEAF. Molecular docking confirmed their stable binding to hub genes (*IL-1β, IL-6, NF-κB, and TNF-α*), with binding energies below –5.0 kcal/mol (Supplementary Fig. 1A). Specifically, each compound was found to form multiple conventional hydrogen bonds and key hydrophobic interactions (e.g., pi-alkyl, pi-pi stacking) with critical amino acid residues within the active sites of these targets (Supplementary Fig. 1B–F). These results indicate that the five compounds likely exert their effects by synergistically targeting key functional residues of the hub gene proteins.

### 3,4-dihydroxyphenylpropionic acid, glycitein, and isorhapontigenin are validated as the main bioactive compounds of WEAF against NAFLD in FFA-stimulated HepG2 and LO2 cells

**Fig. 7:**
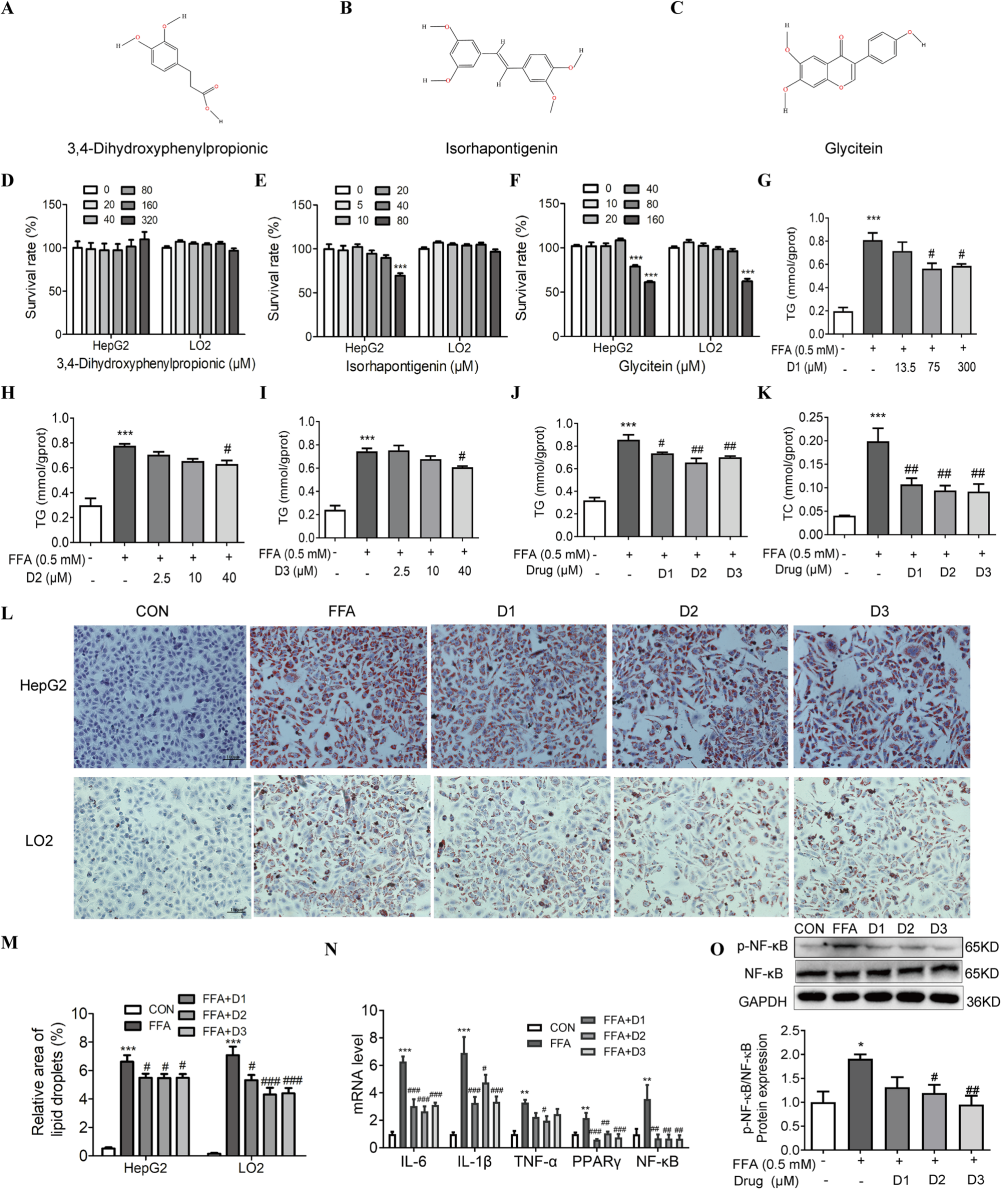
Validation of 3,4-dihydroxyphenylpropionic acid, glycitein, and isorhapontigenin as major bioactive compounds of WEAF against NAFLD in FFA-stimulated HepG2 and LO2 cells. **A** The 2D structure of 3,4-dihydroxyphenylpropionic acid. **B** The 2D structure of isorhapontigenin. **C** The 2D structure of glycitein. **D** Cell survival rate of 3,4-dihydroxyphenylpropionic acid. **E** Cell survival rate of isorhapontigenin. **F** Cell survival rate of glycitein. **G** TG content of 3,4-dihydroxyphenylpropionic acid in HepG2. **H** TG content of isorhapontigenin in HepG2. **I** TG content of glycitein of HepG2. **J** TG content of 3,4-dihydroxyphenylpropionic acid, glycitein, and isorhapontigenin in LO2 cells. **K** TC content of 3,4-dihydroxyphenylpropionic acid, glycitein, and isorhapontigenin in HePG2 cells. **L** Oil red O staining of HepG2 and LO2 cells. (scale bar: 100 μm ×20 magnification). **M** Relative area of lipid droplets. **N** IL-6, IL-1β, TNF-α, PPARγ and NF-κB mRNA expression in HepG2 cells. **O** Protein expression level of p-NF-κB/ NF-κB in HepG2 cells. Mean ± SEM, *n* = 3 for panels (**D**–**M**), *n* = 6 for qPCR analysis (panel **N**), *n* = 5 for Western blot analysis (panel **O**); ***p < 0.001, **p < 0.01 vs. CON group; ^###^*p* < 0.001, ^##^*p* < 0.01, ^#^*p* < 0.05, vs. FFA grou*p*. D1 3,4-dihydroxyphenylpropionic acid (300 μM), D2 Isorhapontigenin (40 μM); D3 Glycitein (40 μM).

Using network pharmacology and molecular docking, we screened five candidate compounds from WEAF (Fig. 7A–C, Supplementary Fig. 2A, B). Their non-cytotoxic concentration range was subsequently determined by CCK-8 assay (Fig. 7D–F, Supplementary Fig. 2C, D). In FFA-stimulated HepG2 and LO2 cells, three compounds—3,4-dihydroxyphenylpropionic acid, glycitein, and isorhapontigenin—demonstrated significant lipid-lowering effects. These active compounds dose-dependently reduced intracellular TG and TC content (Fig. 7G–K, Supplementary Fig 2E, F) and decreased lipid accumulation as shown by Oil Red O staining (Fig. 7L, M), while Luteolin and Methyl cinnamate showed no significant effects on level of TG and TC at non-cytotoxic concentrations (Supplementary Fig. 2E, F). Mechanistic studies revealed that the three active compounds modulated the NF-κB and PPAR-γ signaling pathways. They not only downregulated the mRNA expression of NF-κB, PPAR-γ, IL-1β, TNFα, and IL-6 (Fig. 7N), but also downregulated the protein expression of p-NF-κB/ NF-κB (Fig. 7O). These findings identify 3,4-dihydroxyphenylpropionic acid, glycitein, and isorhapontigenin as the key WEAF constituents that ameliorate NAFLD through NF-κB/PPAR-γ pathway regulation.

### TLR2 is a direct target of glycitein and isorhapontigenin

**Fig. 8:**
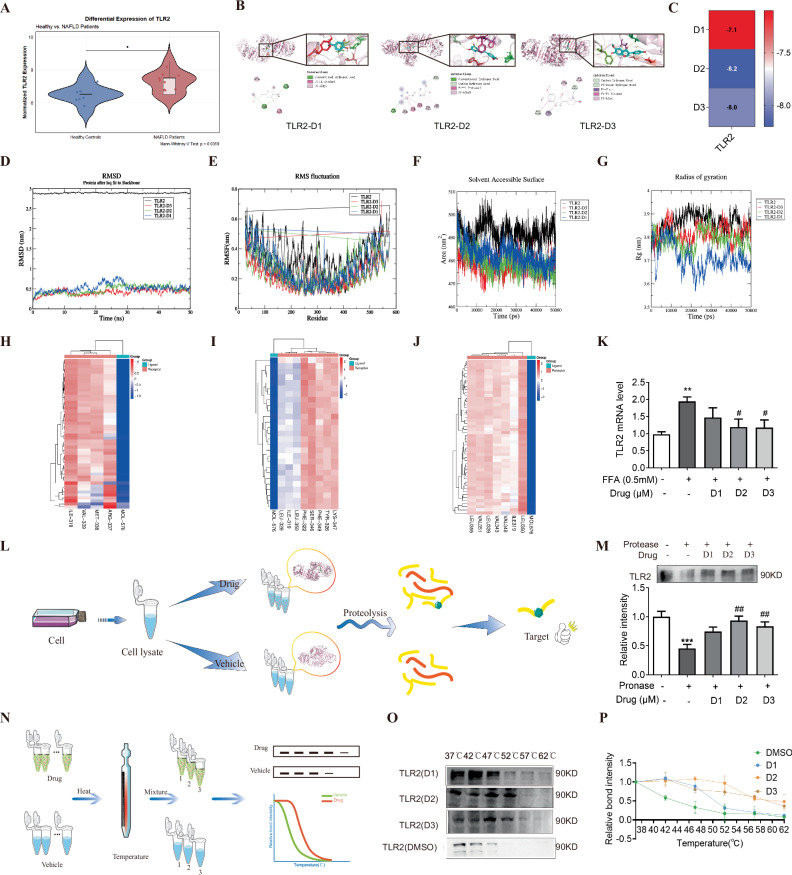
TLR2 as a direct target of glycitein and isorhapontigenin. **A** TLR2 expression levels in liver samples from normal donors (*n* = 7) and NAFLD patients (*n* = 8). **B** Molecular binding of 3,4-dihydroxyphenylpropionic acid (D1), glycitein (D2), and isorhapontigenin (D3) with TLR2. **C** The molecular binding scores of D1, D2, and D3 with TLR2. **D**–**G** Results of root-mean-square deviation (RMSD, **D**), root-mean-square fluctuation (RMSF, **E**), solvent-accessible surface area (SASA, **F**), and radius of gyration (Rg, **G**) for the complexes of D1, D2, D3 with TLR2, analyzed over the molecular dynamics (MD) simulation. **H**–**J** Residual energy decomposition of the MM-PBSA binding energy of the complexes calculated using 30–40 ns MD simulation, indicating the contribution of different residues to the binding energy (D1, **H**; D2, **I**; D3, **G**). **K** Relative mRNA level of TLR2. **L** The flowchart of DARTS. **M** DARTS assay for the interaction between monomers and TLR2. **N** The flowchart of CETSA. **O** CETSA assay for the interaction between monomers and TLR2. **P** Relative bound intensity of CETSA assay. Mean ± SEM, *n* = 6 for RT-qPCR (panel **K**), *n* = 3 for DARTS (panels **M**) and CETSA (panels **O**, **P**); ***p < 0.001, **p < 0.01 vs. CON group; ^#^^#^*p* < 0.01, ^#^*p* < 0.05, vs. FFA group. D1 3,4-dihydroxyphenylpropionic acid (300 μM), D2 isorhapontigenin (40 μM), D3 glycitein (40 μM), MD Molecular dynamics, RMSD Root mean square deviation, RMSF Root mean square fluctuation, Rg Radius of gyration, SASA Solvent accessible surface area, DARTS Drug affinity responsive target stability, CETSA Cellular thermal shift assay.

Transcriptomics analysis of the GEO database showed that the normalized TLR2 expression was higher in the NAFLD group than in the healthy group (Fig. 8A), suggesting that upregulation of TLR2 may contribute to the pathogenesis of NAFLD. Moreover, the results above showed that WEAF strongly regulated the expression of TLR2. Therefore, we supposed that the key bioactive compounds may exert their effects through direct interaction with TLR2, and explored the potential interaction between 3,4-dihydroxyphenylpropionic acid, glycitein, and isorhapontigenin (Fig. 8B) with TLR2 via molecular docking. The results indicated that the three monomers exhibited favorable binding to TLR2, but glycitein and isorhapontigenin demonstrated relatively stronger binding affinity than 3,4-dihydroxyphenylpropionic acid (Fig. 8C).

To further investigate the binding interactions of 3,4-dihydroxyphenylpropionic acid, glycitein, and isorhapontigenin with TLR2, molecular dynamics (MD) simulations—a computational technique for studying the temporal physical movements of atoms and molecules—were performed on the corresponding complexes with TLR2. Throughout the MD simulation, the root mean square deviation (RMSD)—a metric quantifying the average atomic displacement between superimposed structures that reflects simulation system stability—remained below 1 nm for the TLR2-3,4-dihydroxyphenylpropionic acid, TLR2-glycitein, and TLR2-isorhapontigenin systems (Fig. 8D). In contrast, the RMSD of TLR2 was less than 3 nm, strongly suggesting that the small molecules and proteins form a structurally stable complex. Notably, the root mean square fluctuation (RMSF)—a metric quantifying local flexibility along a protein chain by measuring residue deviation from average positions—revealed significantly lower fluctuations in the 100–400 residue range for the complex compared to TLR2 alone, indicating these amino acid residues serve as pivotal binding sites for 3,4-dihydroxyphenylpropionic acid, glycitein, and isorhapontigenin on the TLR2 receptor (Fig. 8E). Furthermore, the variations in the radius of gyration (Rg)—quantifying structural compactness through protein folding tightness, where lower values indicate denser states—and solvent-accessible surface area (SASA), representing the solvent-exposed surface area reflecting conformational changes, were systematically monitored in TLR2 complexes with 3,4-dihydroxyphenylpropionic acid, glycitein, and isorhapontigenin (Fig. 8F, G). The obtained data provide additional evidence that 3,4-dihydroxyphenylpropionic acid, glycitein, and isorhapontigenin maintain a stable binding interaction with TLR2 during the entire course of the simulation.

Subsequently, the binding free energy of simulated trajectories was calculated for three complexes. The results indicated that the binding free energy of the TLR2-3,4-dihydroxyphenylpropionic acid complex was −18.96 kJ/mol, that of the TLR2-isorhapontigenin complex was −12.52 kJ/mol, and the TLR2-glycitein complex exhibited a binding free energy of −24.12 kJ/mol. All these values were negative, suggesting the spontaneous feasibility of the binding reactions. Van der Waals forces (ΔVDWAALS) and electrostatic interactions (ΔEEL) were identified as the major stabilizing forces for all three complexes. Residue-level energy decomposition analysis further revealed specific key residues involved in ligand binding. For the TLR2-3,4-dihydroxyphenylpropionic acid complex, ARG337, MET338, and VAL339 contributed significantly to the binding energy, consistent with the RMSF results, indicating their potential role as critical binding sites. In the TLR2-isorhapontigenin complex, LEU-350, LEU-328, and ILE-319 were the primary residues facilitating binding, while for the TLR2-glycitein complex, ILE-319 and VAL-348 played a pivotal role. These findings were in line with the RMSF data, highlighting these residues as potential key sites for ligand-receptor interactions (Fig. 8H–J).

In terms of expression level, glycitein and isorhapontigenin were able to significantly inhibit the expression of TLR2 (Fig. 8K). After that, DARTS and CETSA experiments were used to validate the direct binding of 3 monomers with TLR2, and the results of DARTS experiments revealed that treatment of glycitein and isorhapontigenin significantly enhanced the resistance of TLR2 to pronase (Fig. 8M). CETSA experiment results also showed that treatment of 3,4-dihydroxyphenylpropionic acid, glycitein, and isorhapontigenin significantly improved the thermal stability of TLR2, but glycitein and isorhapontigenin had a more notable effect (Fig. 8O, P). The above results indicate that TLR2 is a direct target of glycitein and isorhapontigenin. The HPLC analysis demonstrated that glycitein and isorhapontigenin were present in WEAF at concentrations of 1.02% and 1.94%, respectively. These results quantitatively confirm that both glycitein and isorhapontigenin are major bioactive constituents in the WEAF extract, providing the material basis for their observed pharmacological effects in our subsequent biological evaluations (Supplementary Fig 3A–F).

### Activation of TLR2 abolished the effect of WEAF, glycitein, and isorhapontigenin against NAFLD in HepG2 cells via NF-κB/PPAR-γ signaling

**Fig. 9:**
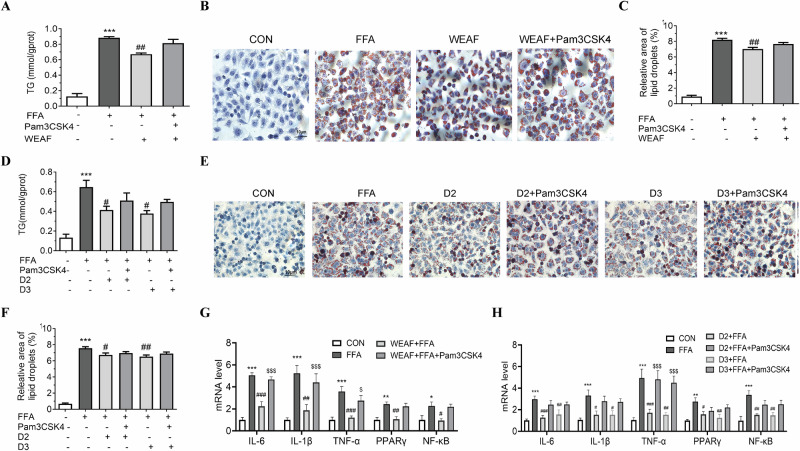
Activation of TLR2 abolished the effect of WEAF, glycitein and isorhapontigenin against NAFLD in HepG2 cells via NF-κB/PPAR-γ signaling. **A** TG content in HepG2 cells treated with WEAF alone or in combination with a TLR2 agonist. **B** Representative Oil red O staining in HepG2 cells treated with WEAF alone or in combination with a TLR2 agonist (Scale bar: 10 μm ×40 magnification). **C** Relative area of lipid droplets in HepG2 cells treated with WEAF alone or in combination with a TLR2 agonist. **D** TG content in HepG2 cells treated with glycitein, and isorhapontigenin alone or in combination with a TLR2 agonist. **E** Representative Oil red O staining images in HepG2 cells treated with glycitein, and isorhapontigenin alone or in combination with a TLR2 agonist (scale bar: ×40 magnification). **F** Relative area of lipid droplets in HepG2 cells treated with glycitein, and isorhapontigenin alone or in combination with a TLR2 agonist. **G** mRNA expression levels of IL-6, IL-1β, TNF-α, PPARγ, and NF-κB in HepG2 cells treated with WEAF alone or in combination with a TLR2 agonist. **H** mRNA expression levels of IL-6, IL-1β, TNFα, PPARγ, and NF-κB in HepG2 cells treated with glycitein, and isorhapontigenin alone or in combination with a TLR2 agonist. Mean ± SEM, *n* = 3 for panels (**A**–**F**), *n* = 6 for RT-qPCR (panels **G**, **H**); ***p < 0.001, **p < 0.01 vs. CON group; ^###^*p* < 0.001, ^##^*p* < 0.01, ^#^*p* < 0.05, vs. FFA group; ^$$$^*p* < 0.001, ^$^*p* < 0.05 vs. WEAF or D2 or D3. D2 isorhapontigenin (40 μM), D3 glycitein (40 μM).

To clarify the role of TLR2 in the anti-NAFLD effects of WEAF, glycitein, and isorhapontigenin, FFA-treated HepG2 cells were co-incubated with TLR2-specific agonist Pam3CSK4 (100 ng/mL; MCE, USA, HY-P1180A) and above compounds for 24 h. WEAF (250 μg/mL) significantly reduced intracellular TG levels, but this was largely nullified by Pam3CSK4-induced TLR2 activation (Fig. 9A). Oil red O staining (Fig. 9B, C), consistent with TG quantification, further validated that TLR2 activation, to some extent, counteracts the anti-NAFLD effects of WEAF, suggesting WEAF’s benefit depends on TLR2 inhibition.

Similarly, glycitein and isorhapontigenin (40 μM each) from WEAF reduced intracellular TG levels, and the effect partially reversed by Pam3CSK4. TG measurements (Fig. 9D) and Oil red O staining (Fig. 9E, F) showed that TLR2 activation attenuated their lipid-lowering capacity. Moreover, in FFA-stimulated HepG2 cells, WEAF, glycitein, and isorhapontigenin downregulated the mRNA expression of NF-κB and PPAR-γ and suppressed the mRNA expression of pro-inflammatory cytokines IL-1β, TNF-α, and IL-6. However, co-treatment with Pam3CSK4 eliminated these regulatory effects (Fig. 9G, H). Collectively, these findings indicate that TLR2 activation counteracts the anti-lipid and anti-inflammatory effects of these compounds, highlighting the crucial role of TLR2 in mediating their therapeutic actions against NAFLD.

## Discussion

NAFLD is the most common liver disease, with a prevalence of 25% around the world^1,24^, and increasingly becomes a heavy global health problem, and can be developed into irreversible liver disease, including cirrhosis and liver cancer if not effectively treated in time^24^. In this study, we found that WEAF and its bioactive compounds ameliorate NAFLD through regulating inflammatory response and lipid metabolism via inhibiting TLR2-mediated MYD88/NF-κB and SREBP1/PPAR-γ signaling.

It has been demonstrated that up to 80% of obese people have varying degrees of NAFLD, and weight loss is conducted to attenuate NAFLD^25^. Studies have shown that excessive fat accumulation in the liver leads to liver cell damage and inflammation, which induces the elevation of liver enzymes (ALT, AST), and the elevation of these is associated with hepatic steatosis and fibrosis^26^. Moreover, Excessive nutrient intake results in the accumulation of TG, FFAs, and TC, and leads to the synthesis and utilization of triacylglycerol abnormalities. It is well known that the deposition of TG as lipid droplets in the hepatocytes is the essential characteristic of NAFLD^27^. In this study, we studied the effect of WEAF against NAFLD in FFA-stimulated HepG2 cell and HFD-fed mice, and found that it not only significantly reduced body weight, epididymal fat weight, abdominal fat weight, and adipocytes size of HFD-fed mice, but also decreased the level of serum AST, ALT and liver TG, TC, and relieved the hepatic lipid droplet and vacuole formation, inflammatory cell infiltration and fibrosis, suggesting that WEAF notably attenuates obesity-induced NAFLD in mice.

Network pharmacology is one of the useful multidisciplinary research tools to predict the potential pharmacological mechanism of drugs by giving insights into the biological network relationship between drug compounds, targets, and diseases^28^, and increasingly applied in TCM research in recent years^29^. In this study, we used UPLC-MS/MS-based network pharmacology for evaluating the bioactive compounds and the potential mechanism of WEAF against NAFLD. Drug-compound-target analysis showed that 3,4-dihydroxyphenylpropionic acid, Luteolin, Methyl cinnamate, Glycitein, and Isorhapontigenin may be the main bioactive compounds of WEAF, and experimental validation showed that 3,4-dihydroxyphenylpropionic acid, glycitein, and isorhapontigenin inhibited TG content and red lipid droplet deposition in FFA-stimulated HepG2 and LO2 cells, suggesting that 3,4-dihydroxyhydrocinnamic acid, Glycitein, and Isorhapontigenin are the main active components of WEAF. Moreover, the PPI network, Go enrichment, and KEGG analysis showed that WEAF may regulate the inflammation response, including the release of TNF, IL-6, IL-1β, and NFKB1, by multiple pathways, including NF-κB and PPAR signaling pathways, suggesting that inflammation and lipid metabolism may be involved in the mechanism of WEAF against NAFLD.

Studies also have shown that hepatic inflammation and lipid metabolism are regarded as the key factors in the development of NAFLD^8,30–33^. Immune mediators, such as IL-1β, IL-6, and TNF-α, induce inflammation and contribute to the development of metabolic diseases, including NAFLD^34^. TLR2 is a member of the TLR family, and TLR2 has been shown to mediate liver inflammation and fibrosis, which in turn promotes the progression of NASH^9^. TLR2 activates the downstream signaling factor MyD88, which ultimately stimulates the expression of NF-κB and promotes the release of inflammatory factors (e.g., IL-1β, TNF-α)^10^. MYD88 has an important role in TLR signaling^35^. TLR2-MyD88 signaling pathway has been activated in NAFLD mice, which exacerbates liver inflammation and fibrosis by regulating macrophage polarization and activating NF-κB signaling^31^. As the network pharmacological results predicted, our in vivo and in vitro study also showed that WEAF not only obviously inhibited the mRNA or protein level of hepatic immune mediators IL-6, IL-1β, MCP-1, and TNF-α, but also downregulated the protein level of TLR2, MYD88, p-IKKβ, and p-NF-κB, suggesting that WEAF, at least in part, retards NAFLD through regulating immune mediators via inhibiting TLR2/MYD88/NF-κB signaling.

It is reported that SOCS3 is not only induced by IL-6/STAT3 signaling, but also, like IL-6, is an NF-κB target gene^36^, and blocking of NF-κB downregulated the expression of SOCS3^37^. Researchers found that SOCS3 expression was initiated by the proinflammatory cytokine TNF-α^38^. Moreover, SOCS3 is up-regulated in the liver of obese mice fed high-fat diet, and overexpression of SOCS3 enhances sterol regulatory element-binding protein (SREBP)-1, the key regulator of fatty acid synthesis in the liver^39^ and TG levels in HepG2 cells^40^. In a normal liver, PPARγ is expressed at low levels, but increased expression of PPARγ is regarded as a common characteristic of hepatic steatosis^41^. SREBP1 performs a role in activating PPARγ to regulate lipid synthesis^42^. Hepatic SREBP-1c is activated by inflammatory cytokines and subsequently it activates the downstream gene ACC-1^43^. Consistent with previous research, our network pharmacology results indicated that PPAR signaling is one of the main pathways of WEAF against NAFLD. We also found that WEAF decreased the liver mRNA or protein expression of SOCS3 and PPAR-γ signaling-related markers SREBP1, PPAR-γ, and ACCs in in vivo or in vitro NAFLD models, suggesting that WEAF attenuates the liver lipid disorder and TG content via regulating SREBP1/PPARγ signaling.

Target identification is crucial for drug screening and development because it reveals the mechanism of drug action and ensures the reliability and accuracy of the results^44^. Current approaches for target identification encompass a range of techniques, such as molecular docking, molecular dynamics, DARTS, and CETSA. Molecular docking and molecular dynamics are potent tools for expeditiously discovering drug targets. The drug affinity responsive target stability (DARTS) method, as an innovative strategy in target discovery, showcases distinct advantages in screening small-molecule (SM) targets. Notably, it can perform label-free target screening by exploiting small molecule-induced protein stabilization against proteolysis. The cellular thermal shift assay (CETSA) is a label-free target identification technique. CETSA was used to detect thermal stabilization of target proteins upon ligand binding in intact cells. Both methods serve as complementary biochemical approaches for confirming direct target research^44,45^. In this study, transcriptomic analysis of the GSE63067 dataset from the Gene Expression Omnibus (GEO) database revealed a striking upregulation of TLR2 in NAFLD samples compared to healthy controls, establishing TLR2 as a potential driver of NAFLD pathogenesis^46^. This observation aligns with TLR2’s known role in promoting hepatic inflammation and lipid metabolism dysfunction. To explore therapeutic interventions targeting TLR2, molecular docking and dynamics were employed, identifying 3,4-dihydroxyphenylpropionic acid, Glycitein, and Isorhapontigenin as potential TLR2 regulators with high binding affinity, and we further investigated DARTS and CETSA found that Glycitein and Isorhapontigenin strongly bound and inhibited the expression of TLR2 and increased resistance to protease hydrolysis and thermal stability of TLR2, indicating that the attenuated NAFLD via targeting TLR2. To further validate this mechanism, the TLR2 agonist Pam3CSK4 was employed^47^. When FFA-treated HepG2 cells were co-incubated with Pam3CSK4 and Glycitein or Isorhapontigenin, the anti-NAFLD effects of these compounds were significantly abolished. This finding strongly supports that Glycitein and Isorhapontigenin exert their anti-NAFLD effects via binding TLR2.

Future research should focus on several promising directions to further elucidate the therapeutic mechanisms of WEAF. First, although we identified and validated key individual compounds from the extract, the potential additive or synergistic effects of combining D1, D2 and D3 remain unclear. Second, network pharmacology suggested that WEAF might act against NAFLD through multiple pathways, yet it is unknown whether pathways beyond NF-κB and PPAR also contribute significantly. Third, the involvement of TLR2, a key target, should be validated via TLR2-silenced or knockout models in future studies.

**Fig. 10:**
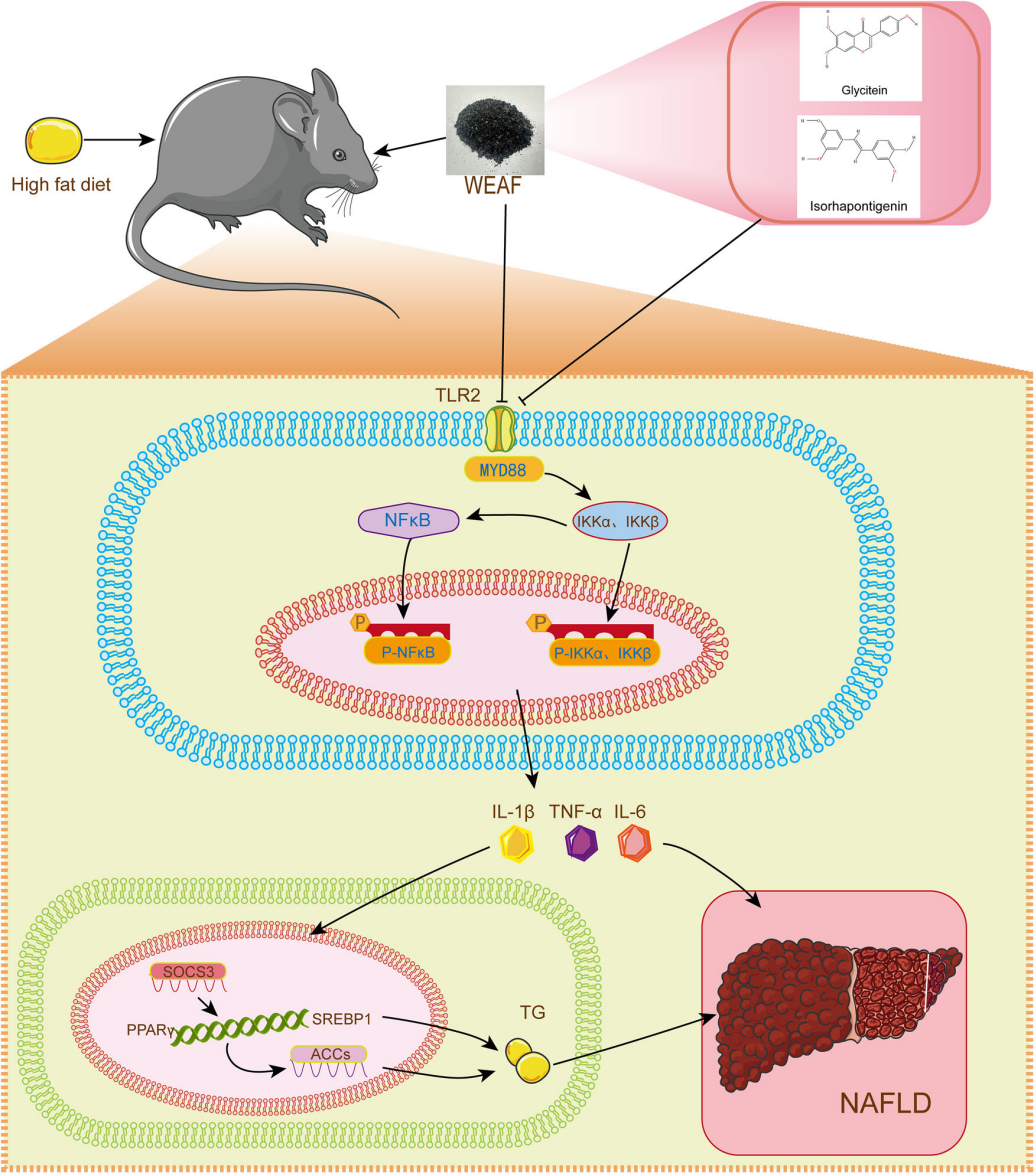
Schematic diagram of the proposed mechanisms underlying the anti-NAFLD effect of WEAF in obese mice. WEAF and its main active compounds alleviate NAFLD-induced inflammatory factors IL-6, IL-1β, and TNF-α through regulating TLR2/MYD88/NF-κB pathway, thereby regulating lipid metabolism-related SREBP1/PPAR-γ signaling, and reducing TG content and fatty acid formation.

Overall, our study demonstrates, for the first time, that WEAF significantly alleviates HFD-induced NAFLD in mice through ameliorating hepatic lipid metabolism disorders, lipid droplet accumulation, liver injury, and hepatitis. As illustrated in Fig. 10, WEAF and its active compounds, Glycitein and Isorhapontigenin, directly targeting TLR2 alleviate NAFLD by mitigating the expression of inflammatory factors (IL-6, IL-1β, and TNF-α) through the regulation of the MYD88/NF-κB and SREBP1/PPAR-γ pathways, concurrently reducing TG content and fatty acid synthesis. Therefore, our findings indicate that WEAF, Glycitein, and Isorhapontigenin as novel therapeutic candidates with promising potential for NAFLD treatment.

## Methods

### Preparation of WEAF

5 kg dried ground part of *Ajania fruticulosa*, verified by Professor Yan Ping in Shihezi University, were chopped and ground into coarse powder, and soaked for 24 h and extracted three times in double-distilled water, with the material-liquid ratio of 1:20, and then filtered, rotary evaporation, and freeze-drying were carried out after mixing the three times of extracts. Finally, we obtained the water extract of *Ajania fruticulosa* (WEAF) and stored it at −20 °C for further experiments.

### UPLC-MS/MS analysis

Solution of WEAF was prepared at a concentration of 1 mg/ml with 80% methanol. After centrifugation of the sample solution at 12,000 rpm for 15 min at 4 °C, the supernatant was collected for further analysis. Analyses of all samples were performed on an ACQUITY Premier UPLC system (Waters, Acquity, USA). The separation was conducted on an ACQUITY UPLC HSS T3 column (2.1*100 mm 1.8 μm) at 40 °C with the flow rate of 0.3 ml/min. Principal component analysis (PCA) was performed on the UPLC-MS/MS data derived from Compound Discoverer 3.3 software.

### Cell culture

HepG2 (human hepatoblastoma cells, HepG2) were donated by the laboratory of Mei Zhang’s team at Shihezi University, and LO2 (human normal liver cells, LO2) were donated by the laboratory of Shenghui Chu’s team at Shihezi University. Both HepG2 and LO2 cells were cultured in RPMI-1640 medium (Biological Industries, Beit-Haemek, Israel) supplemented with 1% penicillin-streptomycin (Solarbio Science Technology, Beijing), HepG2 cells were supplemented with 10% FBS (Biological Industries, Beit-Haemek, Israel), LO2 cells were supplemented with 15% FBS solution and cultured in a humidified incubator (5% CO_2_, 37 °C).

### Cell viability assay

Cell Counting Kit-8 (CCK 8) (Mice, Xi’an, China) assay was used to measure cell viability. Briefly, HepG2 or LO2 cells were seeded into 96-well plates (1 × 10^5^ cells/mL), when cell density reached 50–60% confluence, cells treated with different extracts of AF (dissolved in 0.1% Dimethyl Sulfoxide (DMSO) in incomplete medium to prepare 6 mg/ml stock solution, then diluted with cell culture medium to working concentrations) 3,4-dihydroxyphenylpropionic acid (dissolved in DMSO to prepare a 300 mM stock solution), Glycitein, Isorhapontigenin, Luteolin and Methyl cinnamate (dissolved in DMSO to prepare a 40 mM stock solution) three times for 24 h. The culture medium was discarded, and CCK-8 reagent was added according to the instructions. After incubation in the incubator for 1–4 h, the absorbance (A) value of each well was determined by a microplate reader and analyzed using GraphPad Prism 9.0.

### Determination of intracellular TG and TC content

Intracellular TG content of HepG2 or LO2 stimulated with 0.5 mM FFA was determined as reported before^48^. Briefly, HepG2 or LO2 cells seeded into 6-well plates (5 × 10^5^ cells/well), when cell density reached 70–80% confluence, cells treated with culture medium as a control group, 0.5 mM FFA as a model group, 0.5 mM FFA + WEAF or bioactive compounds such as Luteolin (Jiangsu Yongjian Pharmaceutical Technology Co., Ltd., China, 102759), Isorhapontigenin (Chengdu Push Bio-Technology Co., Ltd., China, 230313), Glycitein (Jiangsu Yongjian Pharmaceutical Technology Co., Ltd., China, YJ0361), 3,4-Dihydroxyphenylpropionic acid (Sigma-Aldrich, USA, 102601) and Methyl cinnamat (Sigma-Aldrich, USA, 96410) or TLR2 agonist Pam3CSK4 for 24 h. The changes in intracellular TG and TC content were measured according to the kit instructions.

### Animal model and treatment

Male C57 BL/6 mice (weight: 18–22 g) aged 7–8 weeks were purchased from Henan Skebes Bio-technology Co., Ltd (Licence No.: SCXK (Yu) 2020-0005). The experiments were conducted in compliance with the National Institutes of Health guidelines on the use of laboratory animals. The University Animal Care Committee for Animal Research at Shihezi University approved the study protocols. (Approval Number: A2024-008) (Xinjiang, China). The mice were placed in a standard animal room: 12 h dark/light cycle, 25 ± 2 °C, and relative humidity of 65 ± 5% with unrestricted consumption of regular food and water. To establish NAFLD mouse model, mice were fed with high-fat-diet (HFD, Custom High fat Diets with Added Leucine, #D12492, Research Diets) or regular diet for 12 weeks after one week acclimatization, and then mice were divided into six group: Control group, HFD group, HFD+low dose (L-D: 0.5 g/kg) treatment group, HFD+medium dose (M-D:1 g/kg) treatment group, HFD+high dose (H-D: 2 g/kg) treatment group, and HFD+Atorvastatin (ATO: 10 mg/kg, positive control drug) treatment group (*n* = 10/group). Atorvastatin was selected as a positive control due to its well-established efficacy as a lipid-lowering agent and its widespread use in preclinical studies of NAFLD/NASH to benchmark the therapeutic potential of investigational treatments^49,50^. After 4 weeks of WEAF or ATO intervention, the mice were fasted for 8 h and anesthetized with 5% isoflurane for 5 min. Following confirmation of adequate anesthetic depth, blood samples were collected via orbital bleeding, and the animals were subsequently euthanized by cervical dislocation. The treatment doses of WEAF and ATO were selected according to our toxicity experiment or previous reports^51^.

### Biochemical assessments

The levels of serum alanine aminotransferase (ALT), aspartate aminotransferase (AST), liver triglyceride (TG) and total cholesterol (TC), were detected according to the assay kit (Nanjing Jiancheng Bio-Engineering Research Institute Co., Ltd., Nanjing, China) instructions.

### Oil Red O staining

Oil Red O staining was employed to assess the accumulation of intracellular lipid droplets. Three mice were randomly selected from each group, and additional liver tissue samples were processed via OCT embedding and cryosectioning prior to staining. HepG2 cells, LO2 cells, or liver tissue sections (5 μm) were first fixed in 10% formalin for 15 min, followed by a brief rinse with 60% isopropyl alcohol for 5–15 s. The samples were then stained with an Oil Red O working solution, prepared by mixing the dye and diluent in a 3:2 ratio, for 20–25 min at room temperature in the dark. After staining, the samples were washed with PBS. Subsequently, the cells and liver slices were incubated in hematoxylin for 1–2 min, followed by a rinse with 60% isopropyl alcohol to remove excess dye, and then washed again with PBS. Finally, the tissue sections were mounted using glycerol gelatin and examined under a microscope (Thermo Fisher Scientific, USA). Intracellular lipid content was quantified using ImageJ software.

### H&E staining and Masson staining

Three mice were randomly selected from each group. Liver tissue was minced and fixed in 10% formalin and embedded in paraffin. Liver sections (5 μm) were dewaxed and rehydrated, stained with hematoxylin and eosin (H&E) or Masson trichrome. The slides were viewed, and images were captured under a light microscope (Carl Zeiss axio imager m2 German), and the NAFLD activity score was evaluated in a blinded manner according to the methods by Kleiner^52^.

### Network pharmacological analysis

The main compound of WEAF was detected by UPLC/MS/MS, and the chemical structure of that compound was obtained from the PubChem database (https://pubchem.ncbi.nlm.nih.gov/, accessed 03/01/2024). The potential targets of WEAF were obtained from TCMSP (http://tcmspw.com/index.php, accessed 04/01/2024), Swiss Target Prediction (http://www.swisstargetprediction, accessed 04/01/2024), CTD (https://ctdbase.org/, accessed 04/01/2024), BATMAN (http://bionet.ncpsb.org.cn/batman-tcm/index.php, accessed 05/01/accessed 2024), and ETCM (http://www.tcmip.cn/ETCM/, accessed 05/01/2024) database. Disease targets for non-alcoholic liver disease were collected from the Gencard (https://www.genecards.org/, accessed 08/01/2024), Home-OMIM (https://www.omim.org, accessed 08/01/2024), PharmGKB (https://www.pharmgkb.org, accessed 09/01/2024), and DisGeNET (https://www.disgenet.org, accessed 09/01/2024). Removal of duplicate targets after the collection of targets for diseases and drugs is complete. The collected target information was imported into the String database (https://cn.string-db.org/, accessed 10/01/2024), and the lowest interaction score greater than 0.7 was selected and saved in TSV format for protein-gene correlations with high confidence. Then, we imported the data into Cytoscape 3.7.0 software and used the Degree value to rank the targets, filtered out the key targets to construct a protein-protein interaction (PPI) network graph of potential targets, and filtered out the top 11 core targets. Using Cytoscape 3.7.0, we still sorted the targets corresponding to each drug according to the Degree value, and constructed and analyzed a “drug-ingredient-target-disease” network diagram based on potential targets. Protein-protein interactions (PPI) were topologically evaluated using the “CytoNCA” tool in Cytoscape 3.7.0 software after being structured in the database STRING. Constructing the regulatory network between key WEAF components and target genes. The core target proteins that were screened by PPI were enriched using the Metascape database. After that, an enrichment analysis using the Kyoto Encyclopedia of Genes and Genomes (KEGG) was then performed in the Metascape online database (https://metascape.org/, accessed 09/02/2024). Compound-disease key targets were uploaded to the DAVID (https://david.ncifcrf.gov/, accessed 10/02/2024) database, analyzed for major biological processes and pathways, and enriched, and the data were downloaded and then analyzed by GO (Gene Ontology) using the microbiology platform (http://www.bioinformatics.com.cn/, accessed 15/02/2024).

### Molecular docking

The PubChem database (https://pubchem.ncbi.nlm.nih.gov, accessed 01/03/2024) furnished the 3D structure of the main active components of WEAF and was saved in pdbqt format using AutoDockTools1.5.7 software. The PDB generated the protein structure. (https://pubchem.ncbi.nlm.nih.gov, accessed 01/03/2024) and saved in pdbqt format using AutoDockTools 1.5.7 software. Protein structures were obtained from the PDB (http://www.rcsb.org, accessed 02/03/2024) database and were searched by “Experimental” and “Homo sapiens”. The protein 3D structures were screened using “Refinement Resolution 1.0-2.0” as the screening condition, and a single chain was preferred. The obtained proteins were processed with DiscoveryStudio2019 software to set the binding site size, and amino acid residues were removed by hydrogenation using AutoDockTools1.5.7 software and saved in pdbqt format. Finally, IL-1β (grid size: x = −5.01882, y = 1.98598, z = 0.722642; box size: 27.6), IL-6 (grid size: x = −31.3609, y = 30.4468, z = 41.4847; box size: 32 Å), NF-κB1 (grid size: x = 19.7973, y = −42.7826, z = −16.3145; box size: 68 Å), TNF-α (grid size: x = 19.2599, y = 12.4488, z = 27.325; box size: 30.2 Å), TLR2 (grid size: x = −19.7277, y = −18.2411, z = 16.188; box size: 61.6 Å) were docked with 3,4-dihydroxyphenylpropionic acid, luteolin, methyl cinnamate, glycitein and isorhapontigenin using vina, and the final docking scores were obtained. Finally, the docking was visualized using DiscoveryStudio2019 and PyMoL 2.5 software.

### Real-time quantitative PCR

Based on the manufacturer’s instructions, total RNA was isolated from cells or liver samples with TRIzol (Sangon Biotech, Shanghai, China, B511321-0100) and cDNA was prepared by using RevertAid First Standart cDNA Synthesis Kit (Thermo Fisher Scientific, Waltham, USA, 91254527). RT-qPCR was performed using StepOnePlus™ RT-PCR System (TransGen Biotech, Beijing, China, #R21019), with the amplification in 20 µl reaction volumes using SYBR green (TransGen Biotech, Beijing, China, MPC2312009-1) and related primers, and the data were analyzed using the^ΔΔ^Ct method. Table 1 lists the primer sequences used in Q-PCR.

**Table 1:** primers sequence

Gene	Sequences
IL-1β	F:CTCGCAGCAGCACATCAACAAG
	R:CCACGGGAAAGACACAGGTAGC
IL-6	F:GGAGCCCACCAAGAACGATAGTC
	R:TCACCAGCATCAGTCCCAAGAAG
TNFα	F:CCACCACGCTCTTCTGTCTACTG
	R:TGGTTTGTGAGTGTGAGGGTCTG
MCP-1	F:ACTCACCTGCTGCTACTCATTCAC
	R:TCTTTGGGACACCTGCTGCTG
SREBP1	F:GCCGAGATGTGCGAACTGGAC
	R:TGTCTTGGTTGTTGATGAGCTGGAG
ACCs	F:ATGCGGCAGGTGCTTGAAGG
	R:ACAGGAACAGTGAGAGGCAATGG
PPARγ	F:GCCAAGGTGCTCCAGAAGATGAC
	R:GTGAAGGCTCATGTCTGTCTCTGTC
NF-κB	F:CAATCATCCACCTTCATTCTCAAC
	R:CCACCACATCTTCCTGCTTAG
TLR2	F:TGATGCTGCCATTATCATTATTATG
	R:CCAGGTAGGTCTTGGTGTTCATTATC

### Western-blotting analysis

The isolation of proteins from liver and cells was performed by using RIPA buffer fortified with protease and phosphatase inhibitors. The concentrations of the protein samples were detected by the BCA Reagent Kit. Protein lysates were separated using SDS-PAGE gels and transferred to a PVDF membrane. After blocking, then immunoblotted with related primary antibodies against TLR2 (1:1000, Abways, CY5102, shanghai, China), MYD88(1:1000, Proteintech, 67969-1-Ig, Wuhan, China), p-IKKβ (1:1000, abclonal, A19606, Wuhan, China), IKKβ (1:1000,Proteintech, 15649-1-AP, Wuhan, China), p-IκBα (1:1000, abclonal, AP1237, Wuhan, China), IκBα (1:1000, Proteintech, 15649-1-AP, Wuhan,China), p-NF-κB(1:1000, Proteintech, 82335-1-RR, Wuhan, China), NF-κB (1:1000, 10745-1-AP, Proteintech, 10268-1-AP, Wuhan, China), ACCs(1:2000, Proteintech, 11491-1-AP, Wuhan, China), PPARγ (1;2000, Proteintech, 16643-1-AP, Wuhan, China), SREBP1(1:1000, Proteintech, 14088-1-AP, Wuhan,China), IL-1β (1:1000, Proteintech, 16806-1-AP, Wuhan, China), Rabbit IgG (1:10000, Proteintech, 30000-0-AP, Wuhan, China), Mouse IgG (1:10000, Proteintech, B900620, Wuhan, China). The protein bands were visualized by chemiluminescent Imager (Shanghai Tanon 5200, China), and ImageJ software was used for analysis.

### Molecular dynamics simulations

For molecular dynamics simulations, the TLR2-target complex that had the top-scoring result in molecular docking was chosen. The simulation was carried out using the GROMACS 2021.5 software package. The OPLS-AA/L force field was applied to the protein. To prepare the small-molecule ligand, the LigParGen service (http://zarbi.chem.yale.edu/ligpargen/) was utilized. The simulation system was configured using the three-point simple point charge (SPC 216) water box model. The MD simulation was conducted within a cubic box, with a 10 Å buffer zone around the protein’s outer surface. To maintain electrical neutrality, an appropriate number of sodium or chloride ions was randomly added. Afterward, NVT and NPT equilibration steps, each lasting 1000 ps, were executed. The protein-ligand complex underwent MD simulation for a maximum of 50 ns. From the generated trajectory, geometric parameters of the system, like the number of hydrogen bonds, root-mean-square deviation (RMSD), and root-mean-square fluctuation (RMSF), were calculated for further study. The binding free energy and per-residue energy decomposition were calculated using the Molecular Mechanics/Poisson–Boltzmann Surface Area (MM/PBSA) method based on the equilibrated trajectories from MD simulations. The energy values (unit: kcal/mol) represent the contribution of individual residues to the overall binding free energy, which was implemented in the GROMACS-compatible tool “gmx_mmpbsa”.

### GEO database mining

Raw data were obtained from the Gene Expression Omnibus (GEO) database (https://www.ncbi.nlm.nih.gov/geo/) by downloading the GSE63067 dataset. The limma 3.50.1 package in the R language was employed to analyze gene expression levels between the Healthy group and the NAFLD group. Prior to analysis, raw data underwent background correction and normalization to eliminate technical variations. The expression data of the target gene TLR2 were then extracted and filtered. Differential expression was defined as |log₂ (fold change)| > 0.5 and adjusted *P* < 0.05. Statistical testing was performed to calculate the fold change of TLR2 expression between the two groups. For visualization, gene expression distributions were plotted using the ggplot2 package, with violin plots comparing TLR2 levels between the Healthy and NAFLD groups. The ggpubr package was used to annotate the statistical significance of intergroup differences (*P*-values).

### Drug affinity responsive target stability assay (DARTS)

HepG2 cells in logarithmic growth phase were taken, washed three times with cold PBS, and collected. The cells were lysed using NP-40 lysis buffer (Cat. N8032, Solarbio, Beijing, China). And the protein content was quantified using the BCA protein quantification kit after lysis was completed. TNC buffer was added to the lysis buffer, and the lysate was homogenized into containers and treated with different concentrations of monomeric drugs or DMSO for one hour at room temperature. At the end of the incubation period, the lysate was digested by adding pronase E (catalog P8360, Solarbio, Beijing, China) to the protein for 20 minutes. Western blotting was then used for further detection.

### Cellular thermal shift assay (CETSA)

Briefly, the collected HepG2 cells were resuspended in PBS, homogenized into 6 aliquots, and transferred into 0.2 mL polymerase chain reaction (PCR) tubes, incubated with different concentrations of monomer drug or DMSO for 2 h. Each sample was heated in parallel for 5 min to the corresponding temperature (range: 37–62 °C). Subsequently, the samples were repeatedly freeze-thawed in liquid nitrogen, centrifuged at 1500 rpm for 15 min at 4 °C, and the supernatant was collected for Western blotting analysis.

### Quantitative analysis of key compounds in WEAF

The quantitative analysis of glycitein and isorhapontigenin in WEAF was performed using a validated high-performance liquid chromatography (HPLC) method with an Agilent 1260 system. Standard compounds (purity >98%) were prepared in methanol for calibration curves. WEAF powder was dissolved in methanol (1.07 mg/mL), followed by vortexing, sonication, centrifugation, and filtration through a 0.22 μm membrane. Chromatographic separation was achieved using an Agilent 5C18A column (4.6 × 250 mm, 5 μm) at 30 °C with a mobile phase of 0.1% formic acid in water (A) and acetonitrile (B) at 1 mL/min, employing a gradient elution. The injection volume was 10 μL with detection at 254 nm.

### Data analysis

The GraphPad Prism program (GraphPad Software, Inc., San Diego, CA, USA) was used to analyze the statistical data in our study. ANOVA, or one-way analysis of variance, was performed to compare the differences among each group. The *P* value of <0.05 was regarded as statistically significant. The mean ± SEM was used to express all data.

## Supplementary information

Supplementary

## Data Availability

We declare that all data relevant to this study are contained in this paper and its supplementary information.
