# Supplementary material for: Computational-experimental study reveals direct target and bioactives of Ajania fruticulosa against NAFLD via TLR2/NF-κB/PPAR-γ signaling
Source: NPJ Sci Food. 2026 Jan 23;10:73. doi: 10.1038/s41538-026-00722-w (PMC12920793; doi:10.1038/s41538-026-00722-w)
Supplement: Supplementary file 1 — Supplementary [file 41538_2026_722_MOESM1_ESM.pdf]

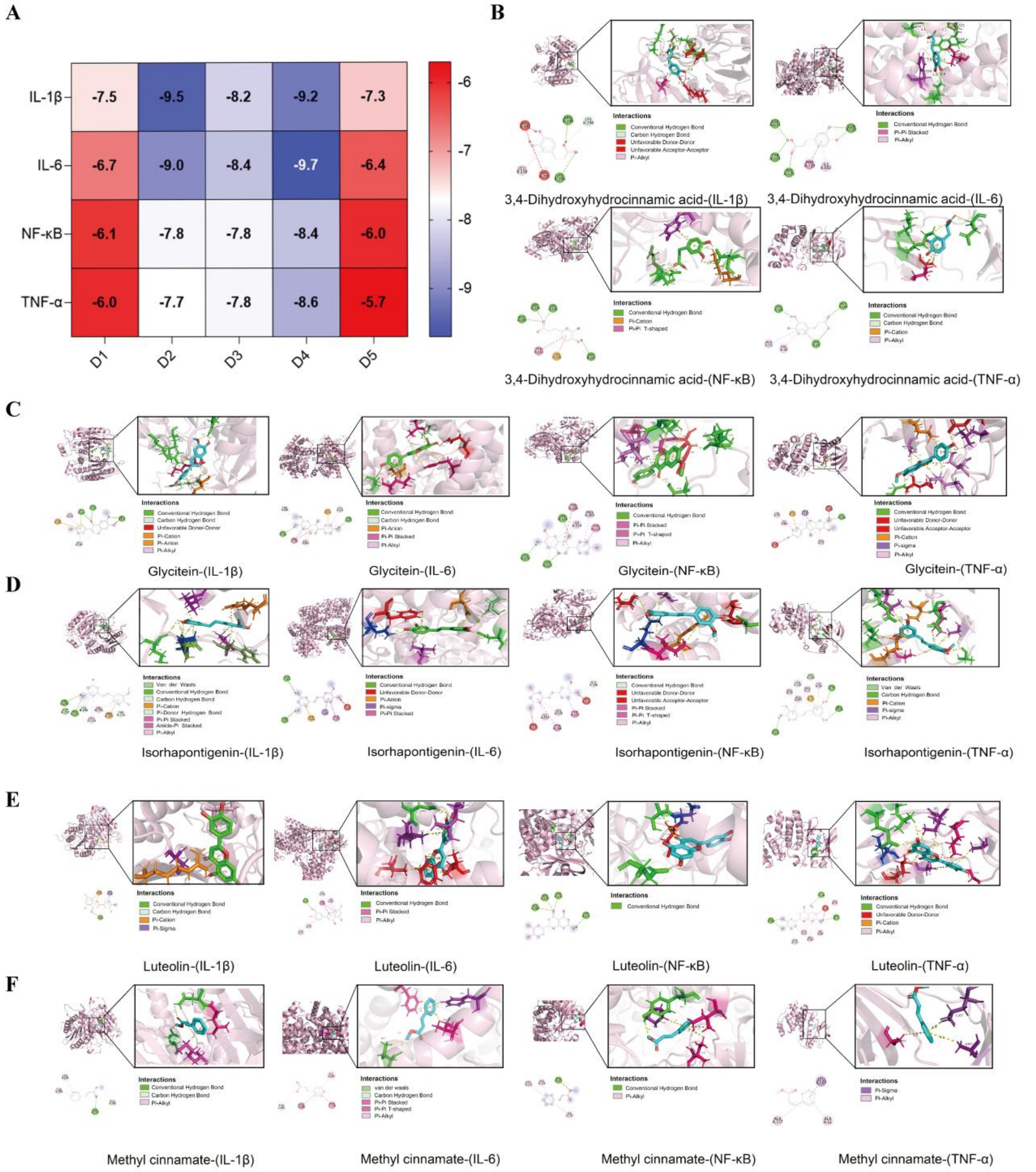

Supplementary Figure 1 | Molecular docking analysis of WEAF-derived compounds with core targets. (A) Docking binding energies of the five bioactive compounds. (B-F) 2D ligand-target interaction diagrams illustrating key molecular bonds for (B) 3,4-Dihydroxyhydrocinnamic acid, (C) Luteolin, (D) Methyl cinnamate, (E) Glycitein, and (F) Isorhapontigenin.

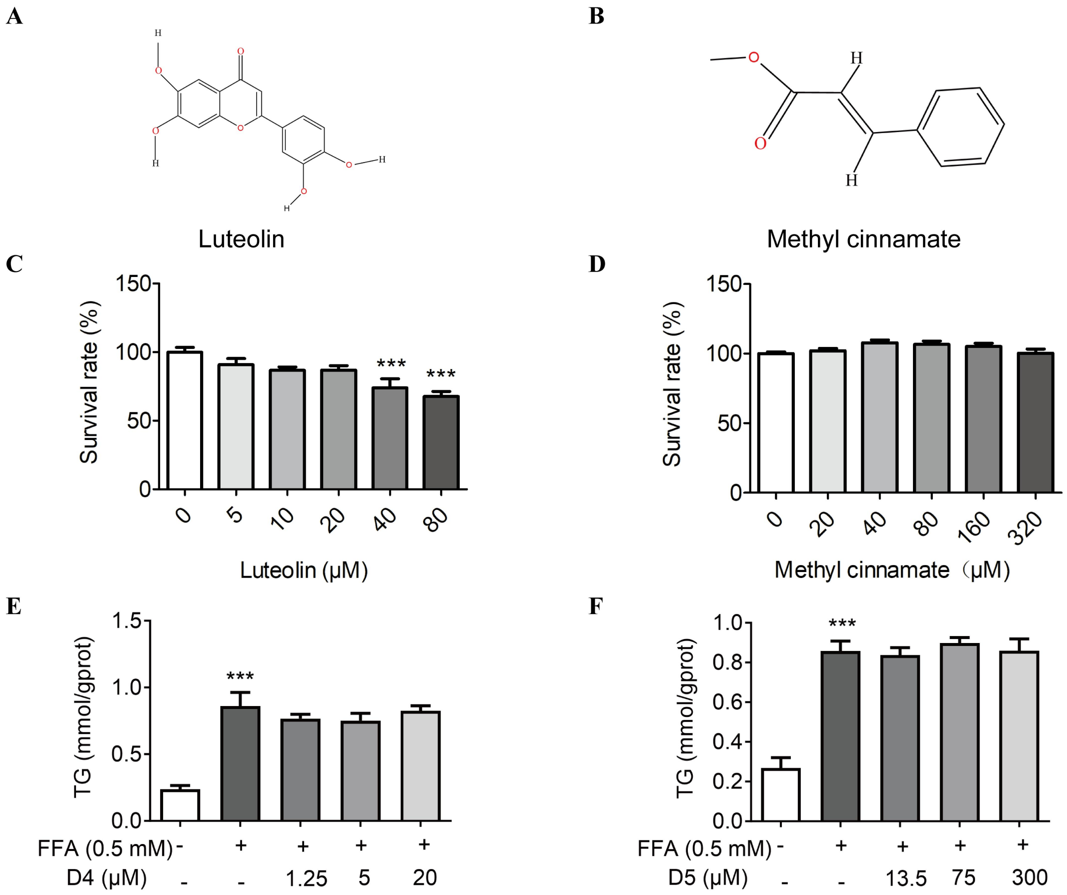

Supplementary Figure 2 | Screening and validation of the candidate compounds from WEAF *in vitro*. (A, B) The chemical structures of the five candidate compounds identified through network pharmacology and molecular docking. (C, D) Cell viability of HepG2 cells treated with the five compounds at various concentrations, as determined by CCK-8 assay, to establish their non-cytotoxic concentration ranges. (E, F) The effects of Luteolin and Methyl cinnamate on intracellular triglyceride (TG) and total cholesterol (TC) levels in free fatty acid (FFA)-stimulated HepG2 cells, showing no significant lipid-lowering activity at non-cytotoxic concentrations. Mean±SEM, n=3, ***p <0.001, *vs* CON group. D4: Luteolin (20 μM); D5: Methyl cinnamate (300μM).

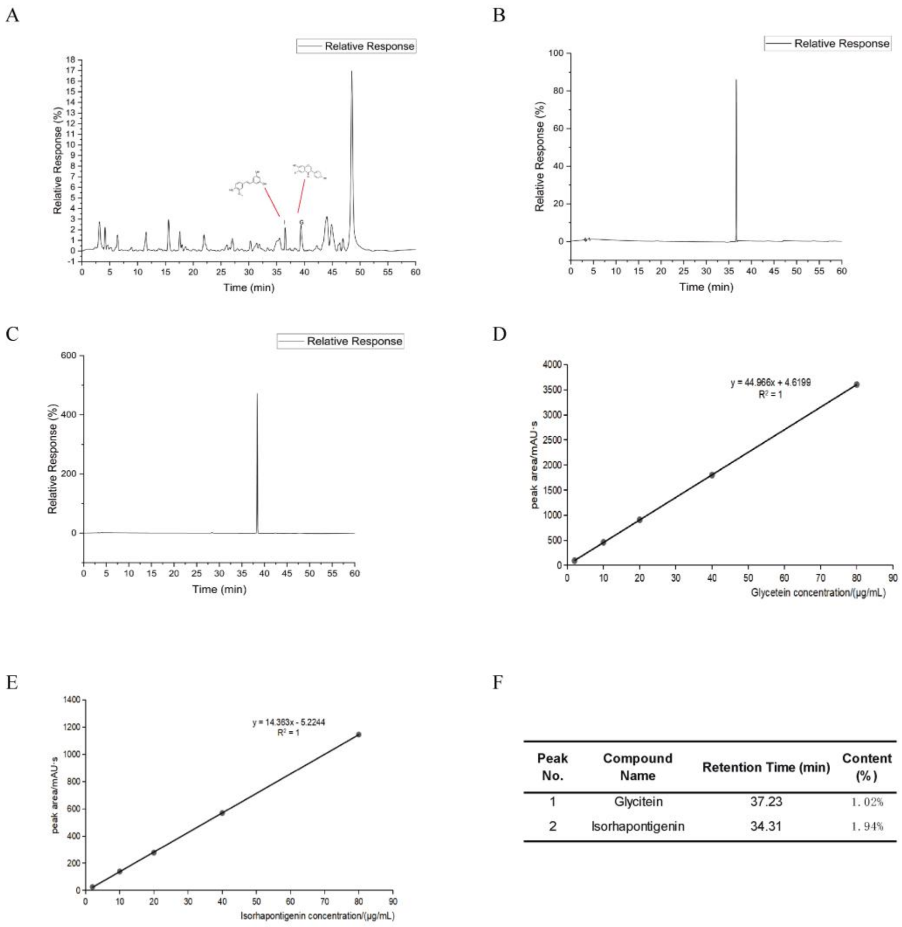

Supplementary Figure 3 | Quantitative analysis of glycitein and isorhapontigenin in WEAF by HPLC. (**A-C**) Representative HPLC chromatograms of (A) the WEAF sample, (B) the glycitein standard, and (C) the isorhapontigenin standard. (D, E) The standard calibration curves for (D) glycitein and (E) isorhapontigenin, used for quantitative determination.

Supplementary Table 1 | Active ingredients in the drug component target map

Degree	name	Compound	Formula
196	W9	Luteolin	C15 H10 O6
94	W2	4-Phenyl-3-buten-2-one	C10 H10 O
91	W16	Azelaic acid	C9 H16 O4
82	W10	Glycitein	C16 H12 O5
65	W3	2-Naphthylamine	C10 H9 N
58	W12	Succinic acid	C4 H6 O4
48	W4	4-Hydroxyphenylacetic acid	C8 H8 O3

48	W13	Gentisic acid	C7 H6 O4
46	W6	Isorhapontigenin	C15 H14 O4
42	W18	Homovanillic acid	C9 H10 O4
38	W19	DL-Tryptophan	C11 H12 N2 O2
32	W15	4-Hydroxybenzoic acid	C7 H6 O3
25	W11	3,4-Dihydroxyphenylpropionic acid	C9 H10 O4
24	W17	2-Hydroxycaproic acid	C6 H12 O3
20	W7	Methyl cinnamate	C10 H10 O2
17	W14	3-Phenyllactic acid	C9 H10 O3
11	W20	Cycloolivil	C20 H24 O7
8	W8	4-Methoxybenzaldehyde	C8 H8 O2
7	W5	2,4-Dimethylbenzaldehyde	C9 H10 O
6	W1	4-Indolecarbaldehyde	C9 H7 NO
1	W21	Genkwanol A	C30H22O10

Supplementary Table 2 | Protein PDB ID

Protein	PDB
IL-1B	3E4C
IL-6	5fuc
NF-κB	5yeu
TNF-α	2a25
TLR2	5d3d

Cell

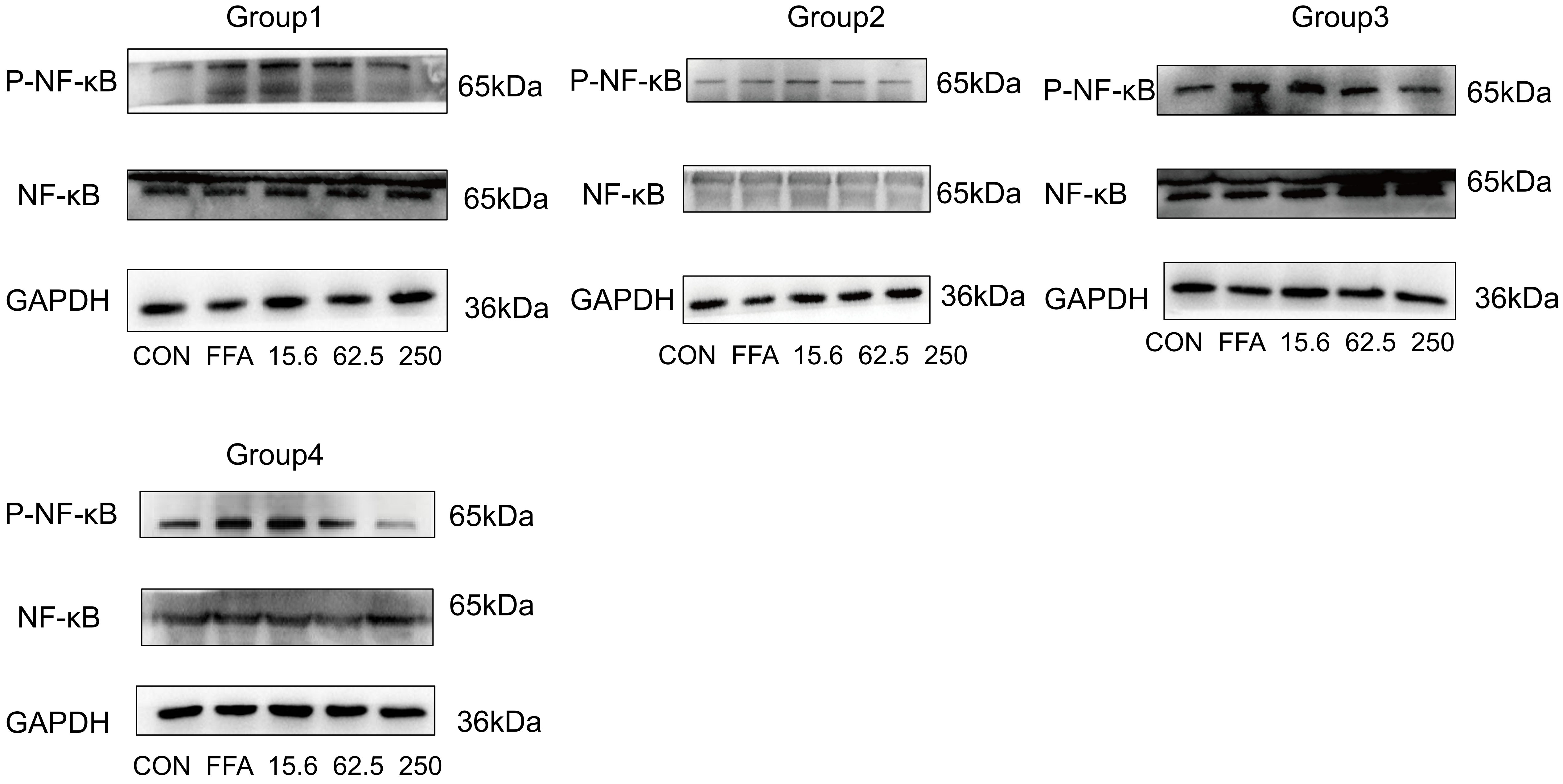
Group1
P-NF-κB
65kDa
NF-κB
65kDa
GAPDH
36kDa
CON FFA 15.6 62.5 250
Group2
P-NF-κB
65kDa
NF-κB
65kDa
GAPDH
36kDa
CON FFA 15.6 62.5 250
Group3
P-NF-κB
65kDa
NF-κB
65kDa
GAPDH
36kDa
CON FFA 15.6 62.5 250
Group4
P-NF-κB
65kDa
NF-κB
65kDa
GAPDH
36kDa
CON FFA 15.6 62.5 250

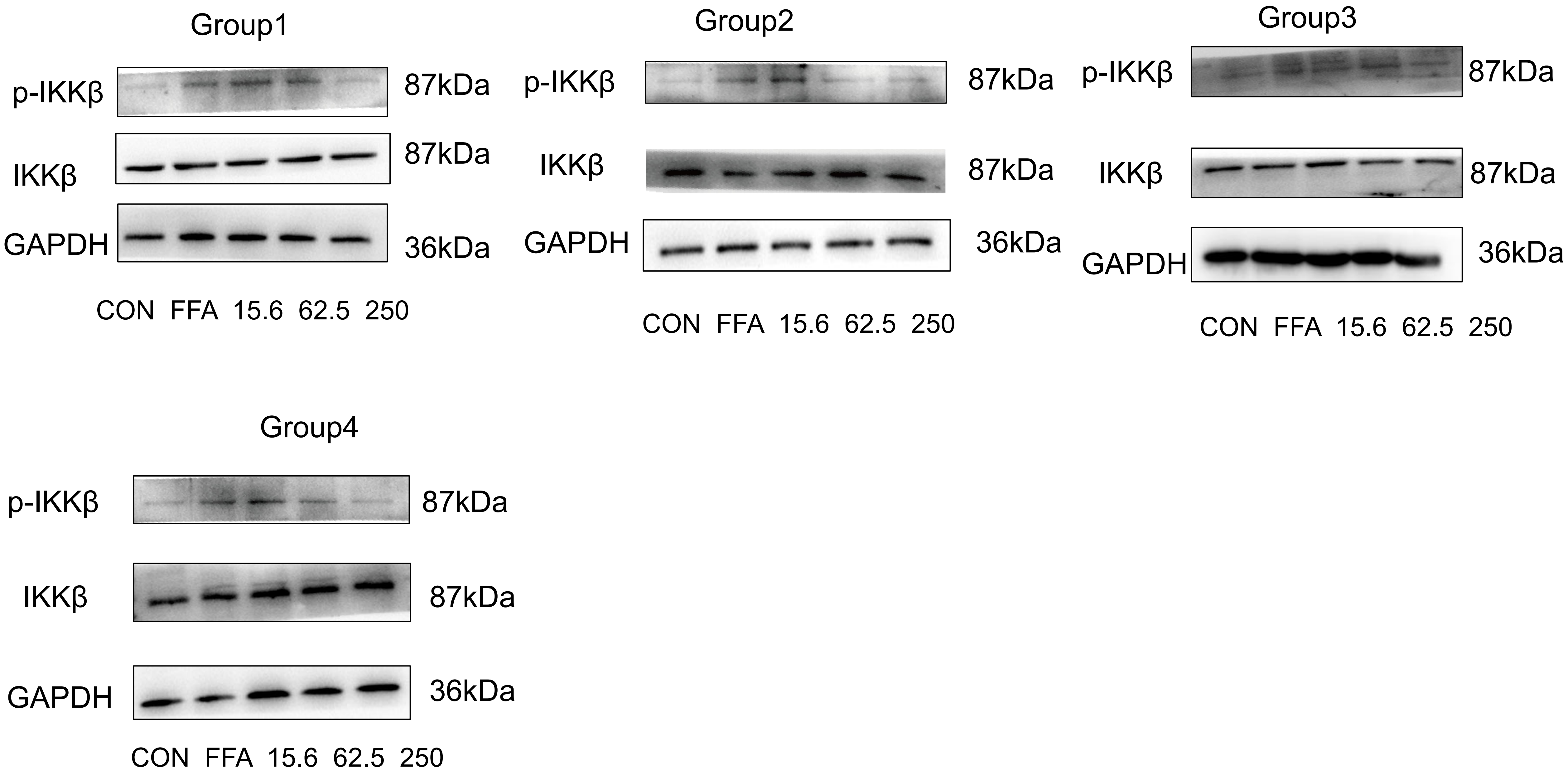
Group1
p-IKKβ
87kDa
IKKβ
87kDa
GAPDH
36kDa
CON FFA 15.6 62.5 250
Group2
p-IKKβ
87kDa
IKKβ
87kDa
GAPDH
36kDa
CON FFA 15.6 62.5 250
Group3
p-IKKβ
87kDa
IKKβ
87kDa
GAPDH
36kDa
CON FFA 15.6 62.5 250
Group4
p-IKKβ
87kDa
IKKβ
87kDa
GAPDH
36kDa
CON FFA 15.6 62.5 250

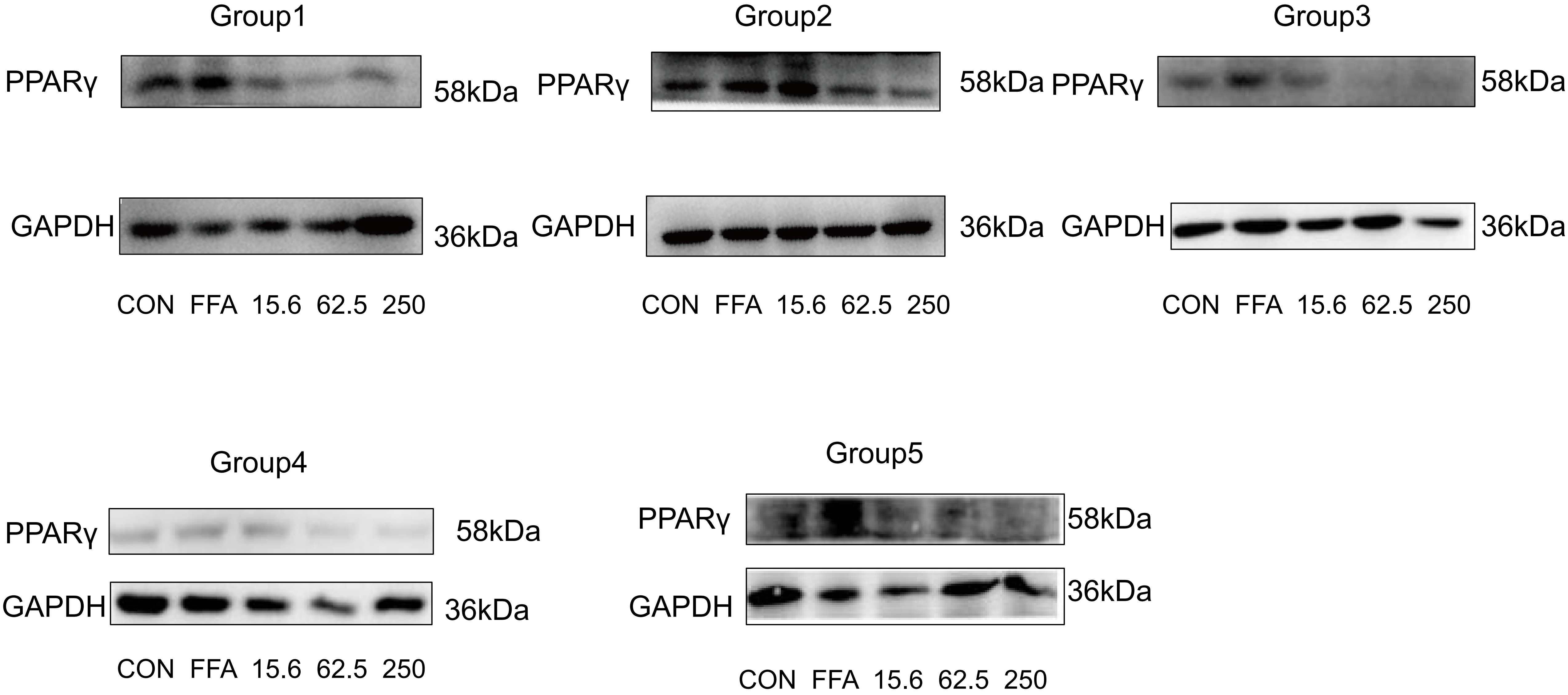
Group1
PPARγ
58kDa
GAPDH
36kDa
CON FFA 15.6 62.5 250
Group2
PPARγ
58kDa
GAPDH
36kDa
CON FFA 15.6 62.5 250
Group3
PPARγ
58kDa
GAPDH
36kDa
CON FFA 15.6 62.5 250
Group4
PPARγ
58kDa
GAPDH
36kDa
CON FFA 15.6 62.5 250
Group5
PPARγ
58kDa
GAPDH
36kDa
CON FFA 15.6 62.5 250

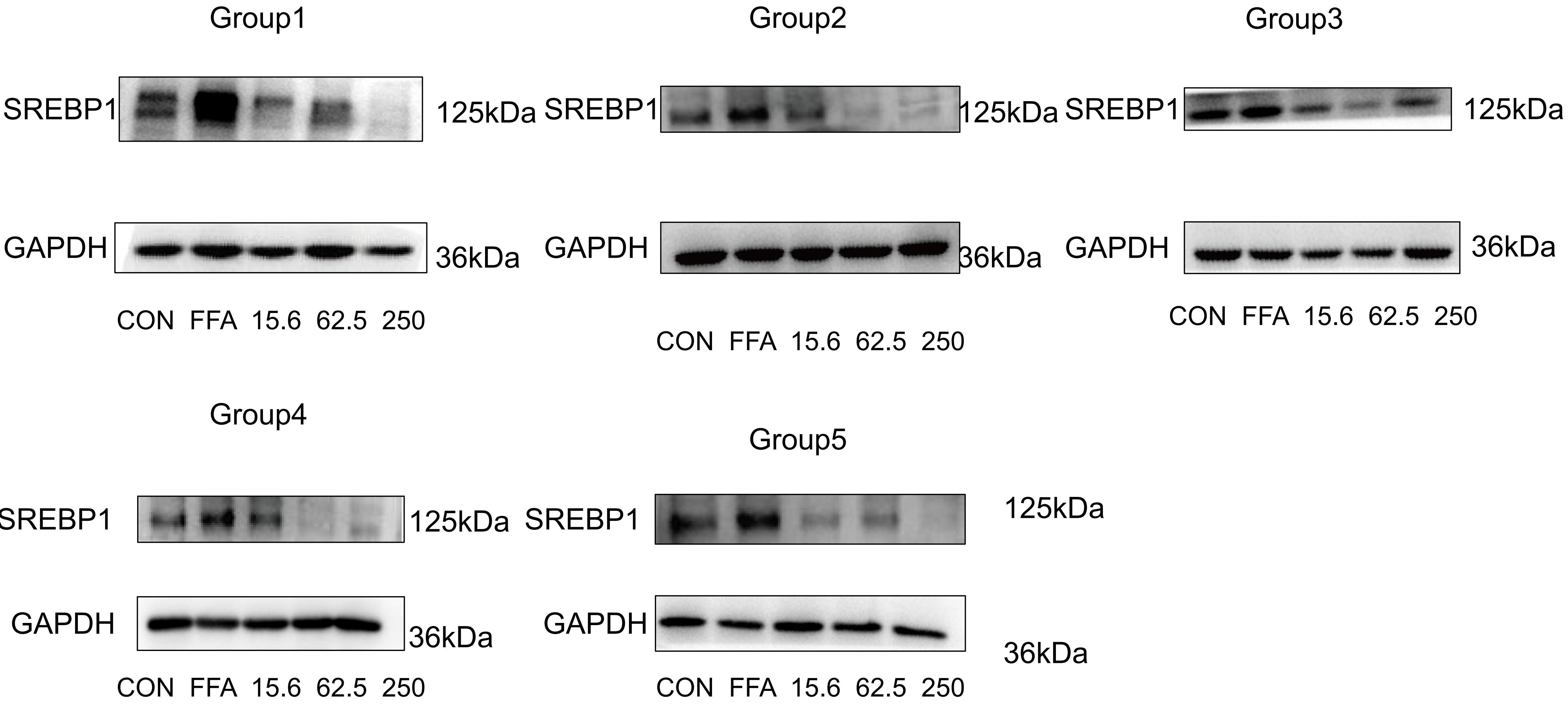
Group1
SREBP1
125kDa
GAPDH
36kDa
CON FFA 15.6 62.5 250
Group2
SREBP1
125kDa
GAPDH
36kDa
CON FFA 15.6 62.5 250
Group3
SREBP1
125kDa
GAPDH
36kDa
CON FFA 15.6 62.5 250
Group4
SREBP1
125kDa
GAPDH
36kDa
CON FFA 15.6 62.5 250
Group5
SREBP1
125kDa
GAPDH
36kDa
CON FFA 15.6 62.5 250

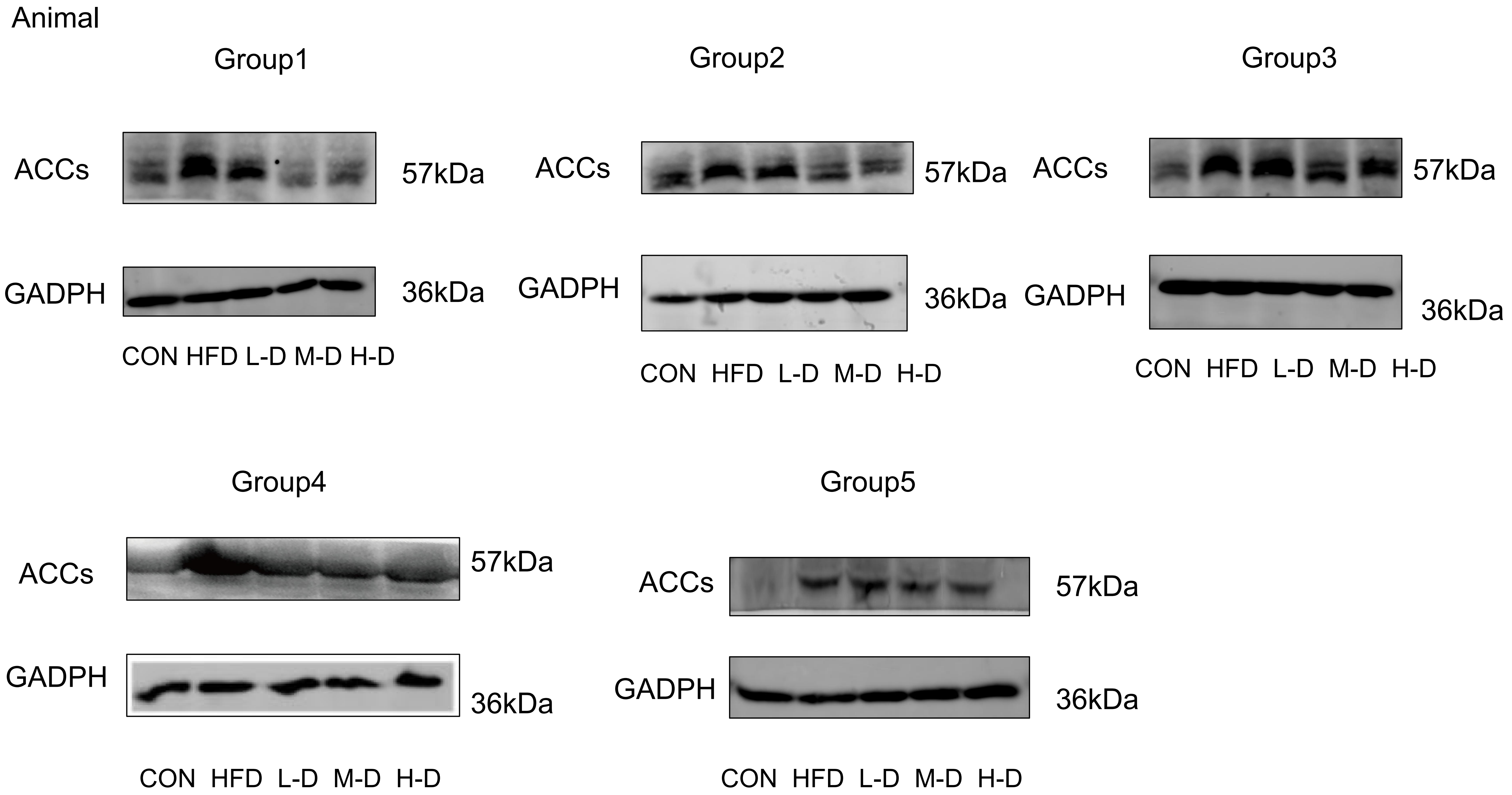
Animal
Group1
ACCs
57kDa
GADPH
36kDa
CON HFD L-D M-D H-D
Group2
ACCs
57kDa
GADPH
36kDa
CON HFD L-D M-D H-D
Group3
ACCs
57kDa
GADPH
36kDa
CON HFD L-D M-D H-D
Group4
ACCs
57kDa
GADPH
36kDa
CON HFD L-D M-D H-D
Group5
ACCs
57kDa
GADPH
36kDa
CON HFD L-D M-D H-D

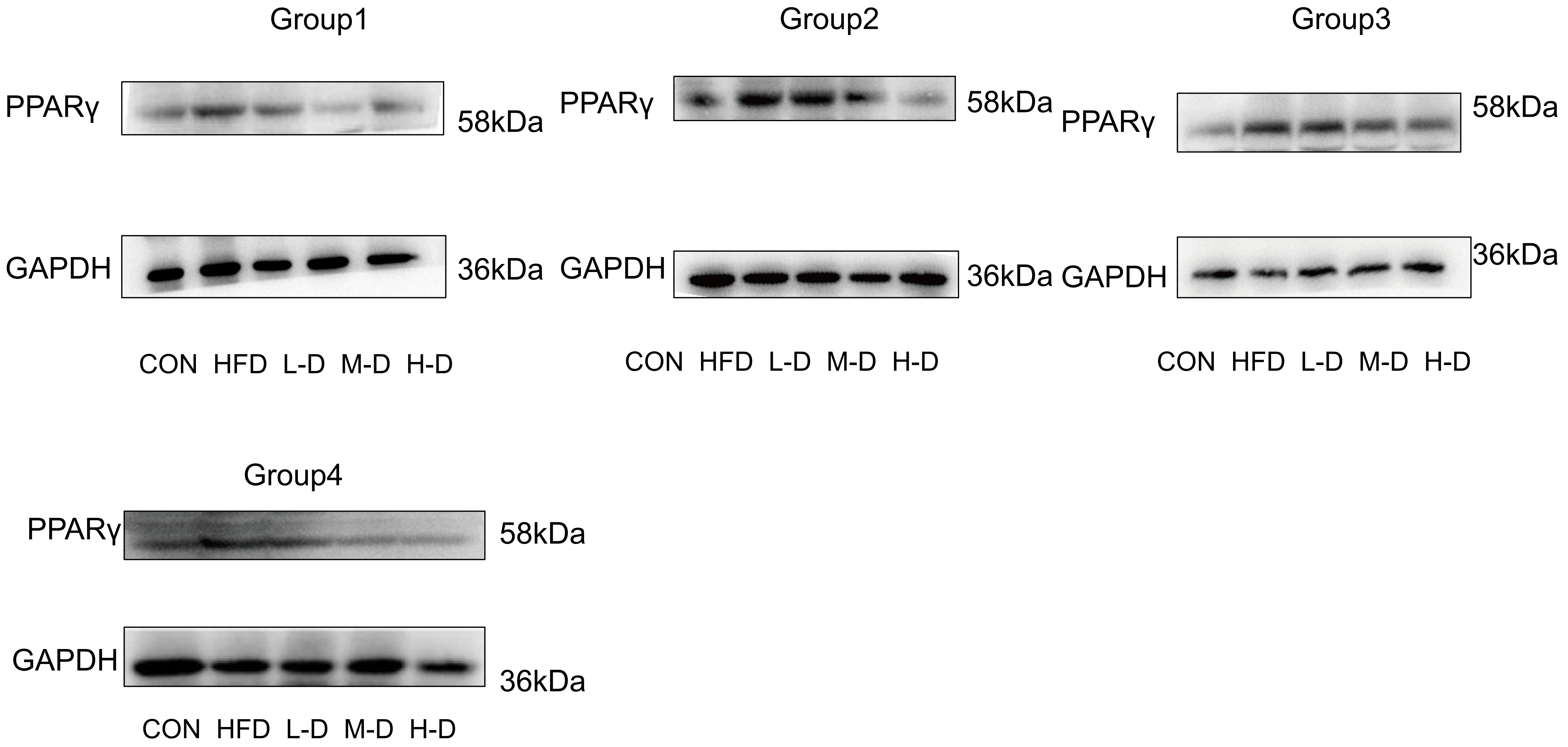
Group1
PPARγ
58kDa
GAPDH
36kDa
CON HFD L-D M-D H-D
Group2
PPARγ
58kDa
GAPDH
36kDa
CON HFD L-D M-D H-D
Group3
PPARγ
58kDa
GAPDH
36kDa
CON HFD L-D M-D H-D
Group4
PPARγ
58kDa
GAPDH
36kDa
CON HFD L-D M-D H-D

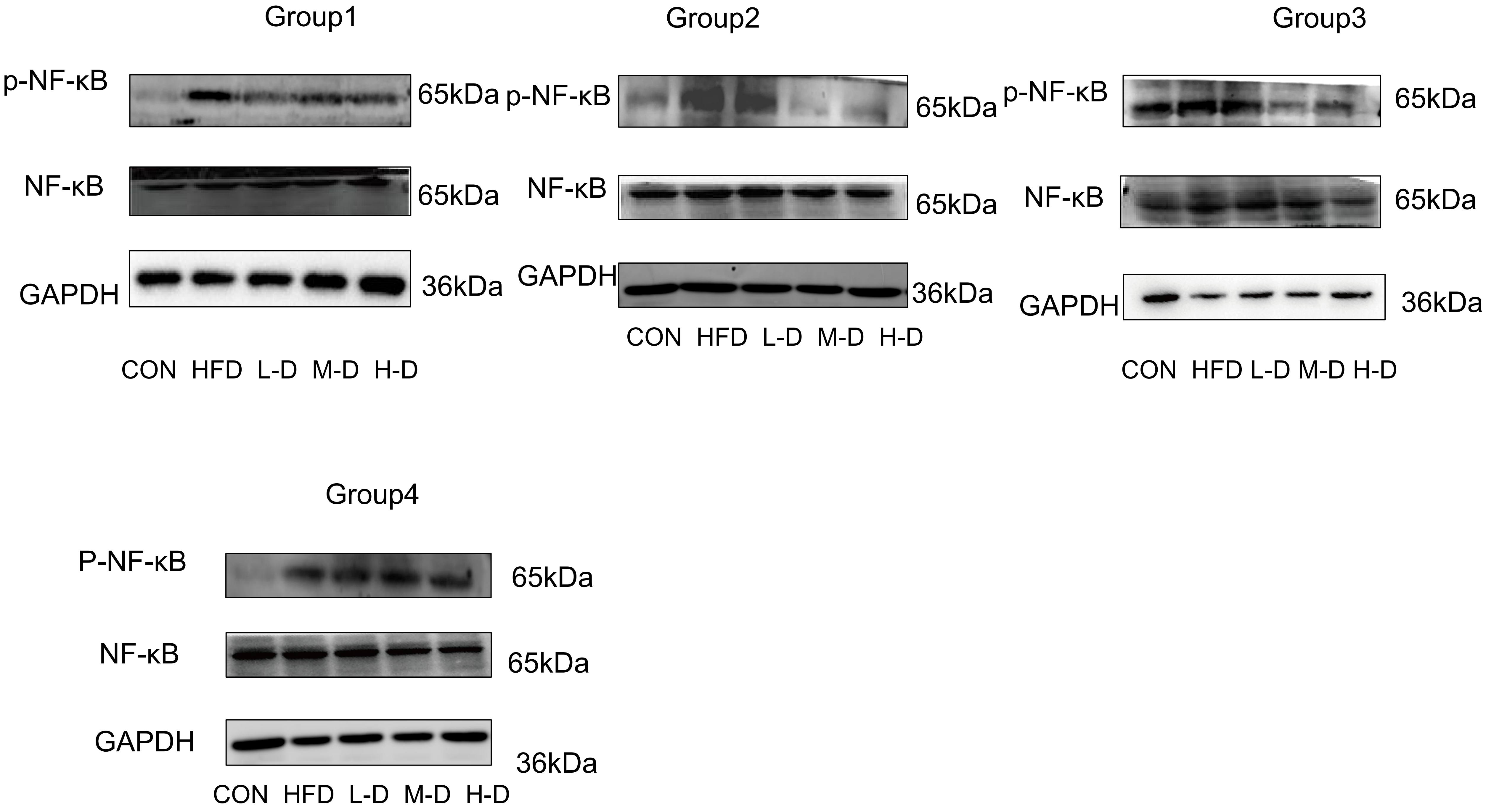
Group1
p-NF-κB
65kDa
NF-κB
65kDa
GAPDH
36kDa
CON HFD L-D M-D H-D
Group2
p-NF-κB
65kDa
NF-κB
65kDa
GAPDH
36kDa
CON HFD L-D M-D H-D
Group3
p-NF-κB
65kDa
NF-κB
65kDa
GAPDH
36kDa
CON HFD L-D M-D H-D
Group4
P-NF-κB
65kDa
NF-κB
65kDa
GAPDH
36kDa
CON HFD L-D M-D H-D

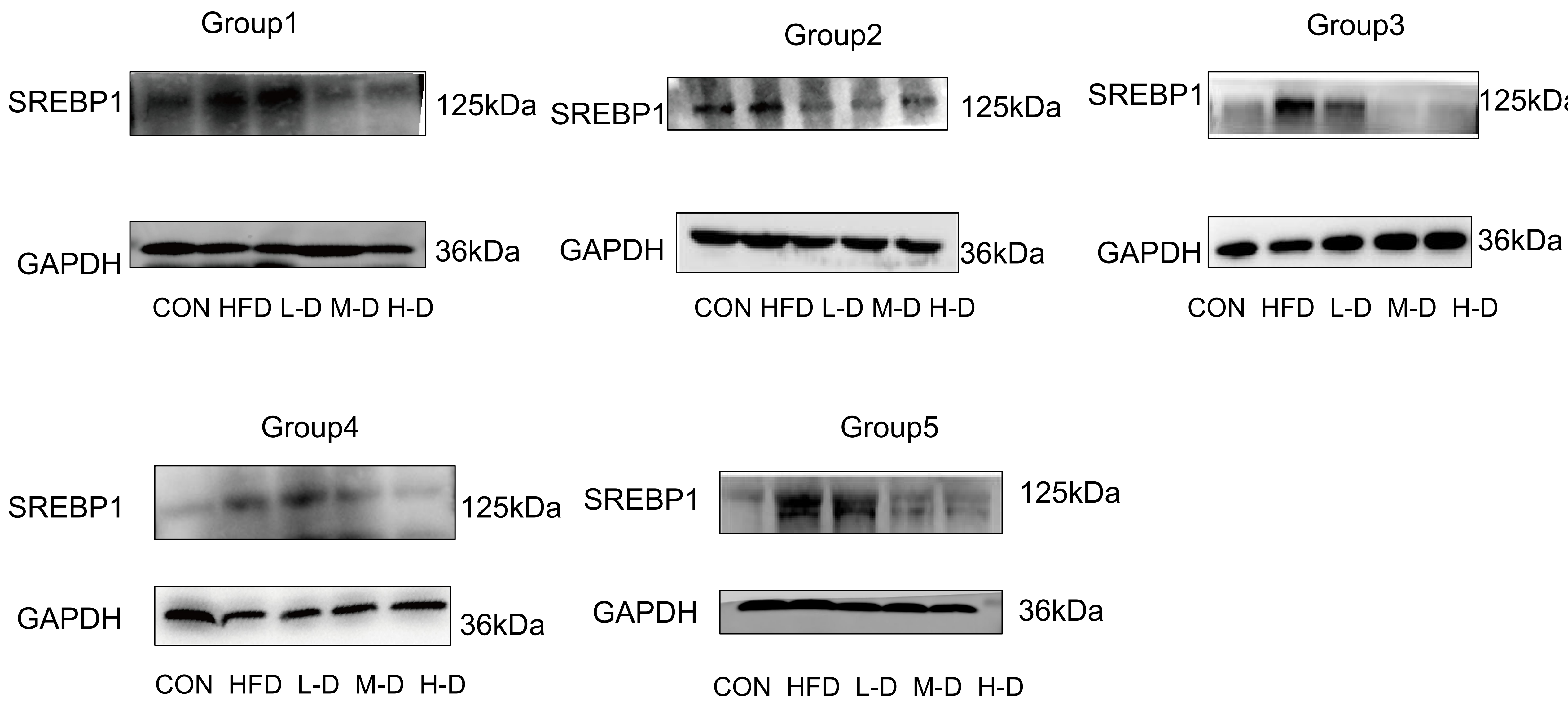
Group1
SREBP1
125kDa
GAPDH
36kDa
CON HFD L-D M-D H-D
Group2
SREBP1
125kDa
GAPDH
36kDa
CON HFD L-D M-D H-D
Group3
SREBP1
125kDa
GAPDH
36kDa
CON HFD L-D M-D H-D
Group4
SREBP1
125kDa
GAPDH
36kDa
CON HFD L-D M-D H-D
Group5
SREBP1
125kDa
GAPDH
36kDa
CON HFD L-D M-D H-D

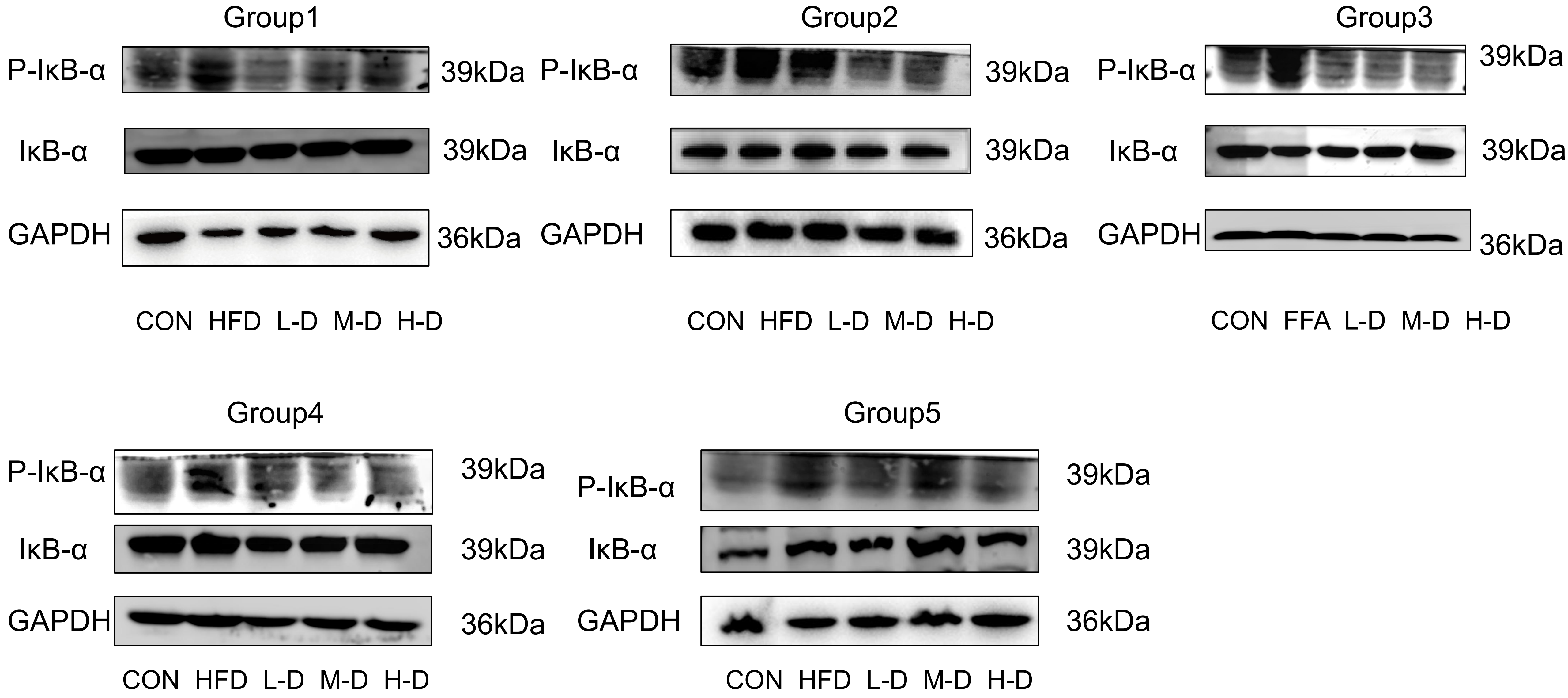

Group1
P-IκB-α
39kDa
IκB-α
39kDa
GAPDH
36kDa
CON HFD L-D M-D H-D
Group2
P-IκB-α
39kDa
IκB-α
39kDa
GAPDH
36kDa
CON HFD L-D M-D H-D
Group3
P-IκB-α
39kDa
IκB-α
39kDa
GAPDH
36kDa
CON FFA L-D M-D H-D
Group4
P-IκB-α
39kDa
IκB-α
39kDa
GAPDH
36kDa
CON HFD L-D M-D H-D
Group5
P-IκB-α
39kDa
IκB-α
39kDa
GAPDH
36kDa
CON HFD L-D M-D H-D

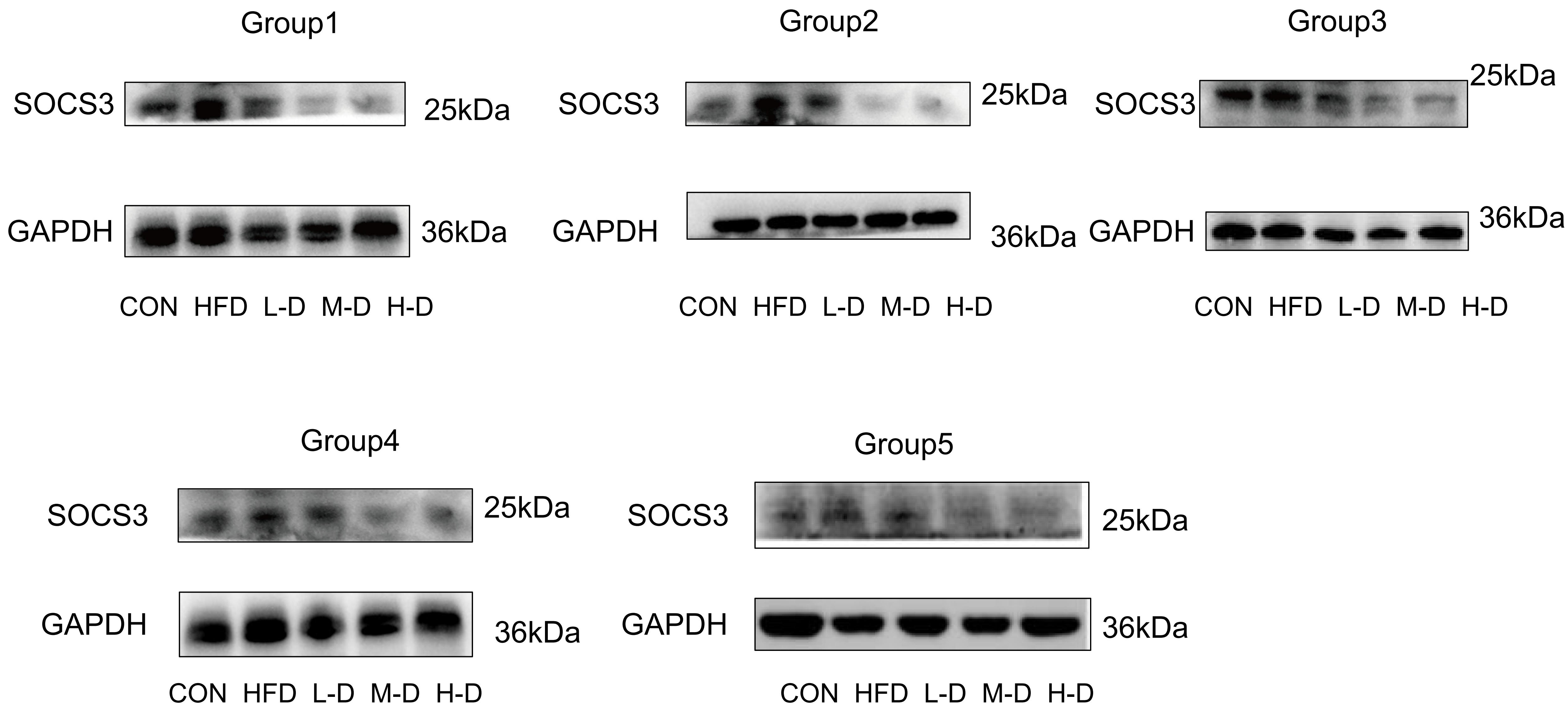
Group1
SOCS3
25kDa
GAPDH
36kDa
CON HFD L-D M-D H-D
Group2
SOCS3
25kDa
GAPDH
36kDa
CON HFD L-D M-D H-D
Group3
SOCS3
25kDa
GAPDH
36kDa
CON HFD L-D M-D H-D
Group4
SOCS3
25kDa
GAPDH
36kDa
CON HFD L-D M-D H-D
Group5
SOCS3
25kDa
GAPDH
36kDa
CON HFD L-D M-D H-D

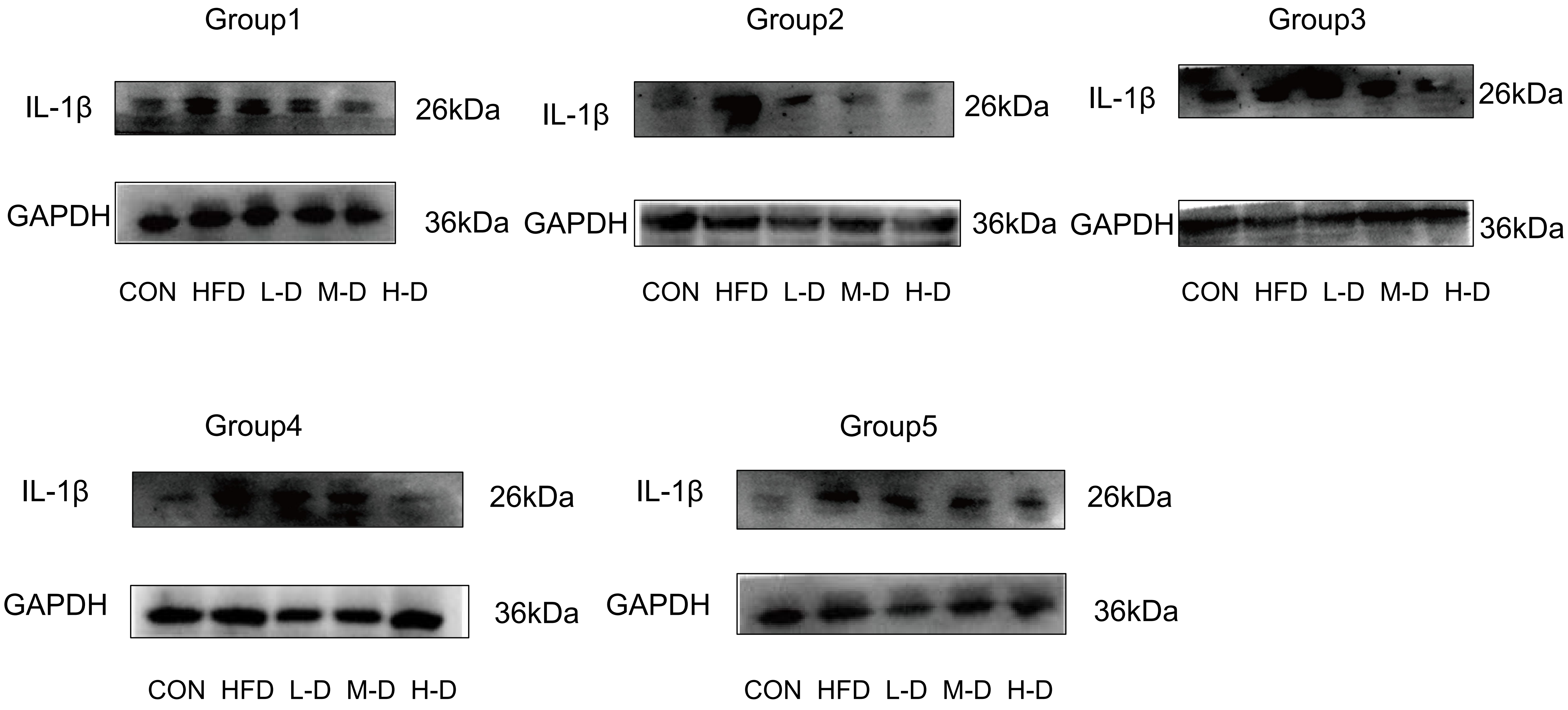
Group1
IL-1β
26kDa
GAPDH
36kDa
CON HFD L-D M-D H-D
Group2
IL-1β
26kDa
GAPDH
36kDa
CON HFD L-D M-D H-D
Group3
IL-1β
26kDa
GAPDH
36kDa
CON HFD L-D M-D H-D
Group4
IL-1β
26kDa
GAPDH
36kDa
CON HFD L-D M-D H-D
Group5
IL-1β
26kDa
GAPDH
36kDa
CON HFD L-D M-D H-D

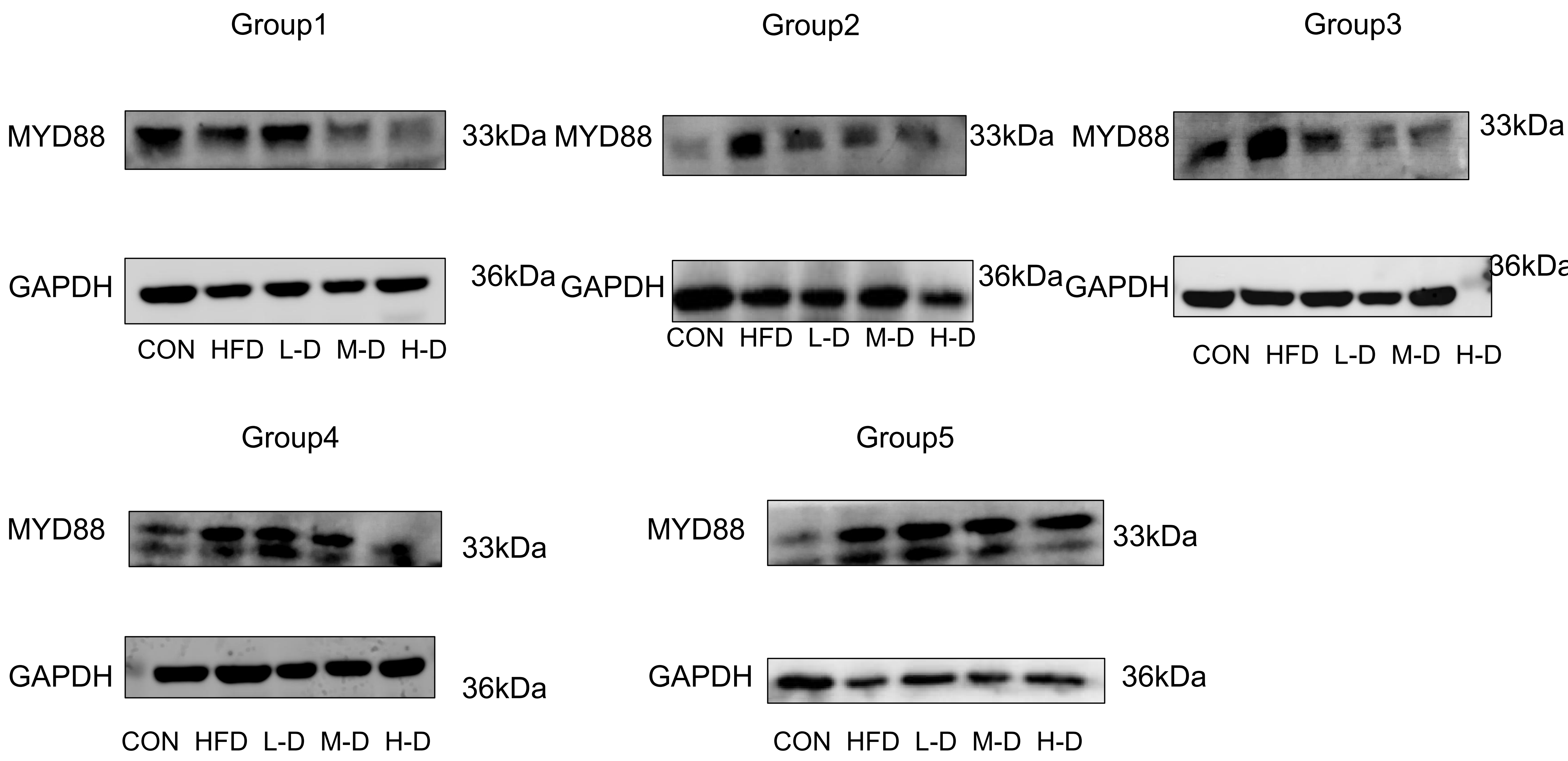
Group1
MYD88
33kDa
GAPDH
36kDa
CON HFD L-D M-D H-D
Group2
MYD88
33kDa
GAPDH
36kDa
CON HFD L-D M-D H-D
Group3
MYD88
33kDa
GAPDH
36kDa
CON HFD L-D M-D H-D
Group4
MYD88
33kDa
GAPDH
36kDa
CON HFD L-D M-D H-D
Group5
MYD88
33kDa
GAPDH
36kDa
CON HFD L-D M-D H-D

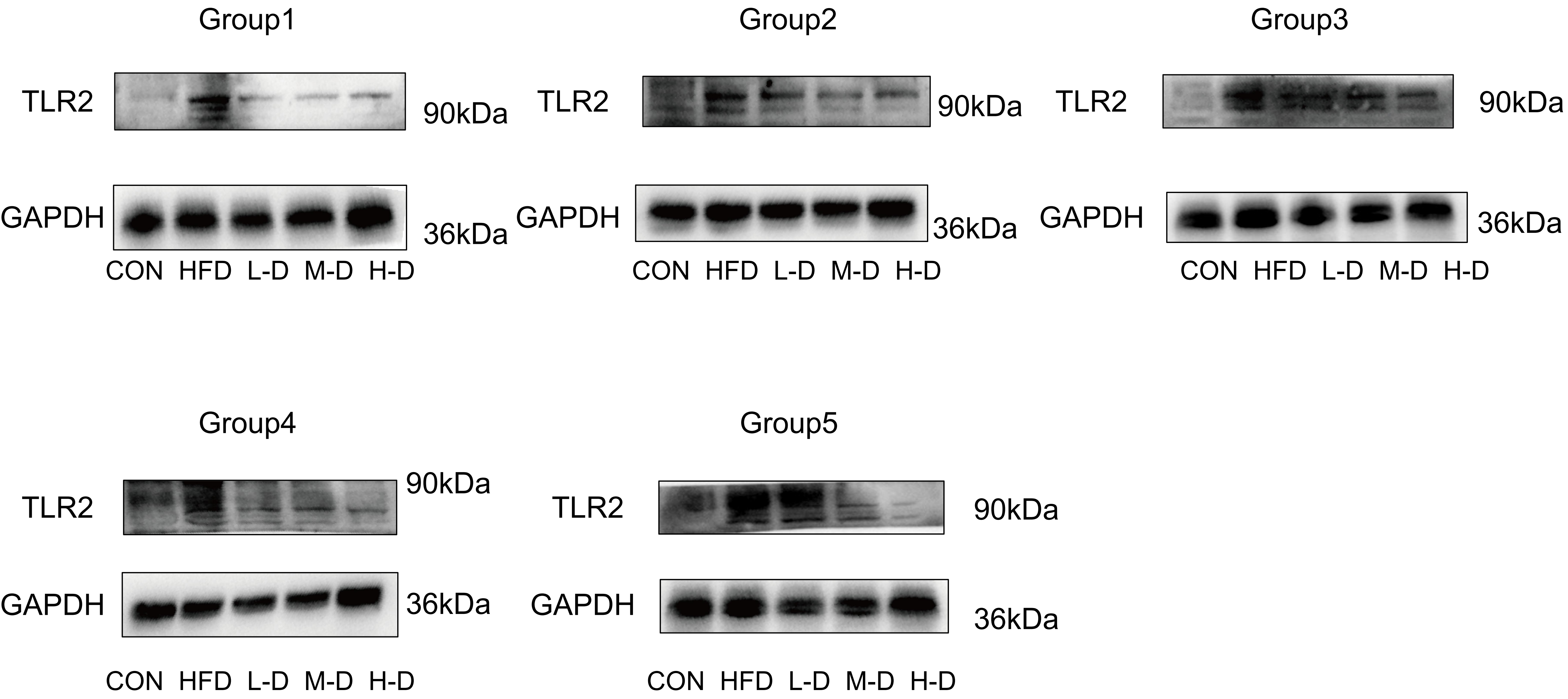
Group1
TLR2
90kDa
GAPDH
36kDa
CON HFD L-D M-D H-D
Group2
TLR2
90kDa
GAPDH
36kDa
CON HFD L-D M-D H-D
Group3
TLR2
90kDa
GAPDH
36kDa
CON HFD L-D M-D H-D
Group4
TLR2
90kDa
GAPDH
36kDa
CON HFD L-D M-D H-D
Group5
TLR2
90kDa
GAPDH
36kDa
CON HFD L-D M-D H-D

Group1

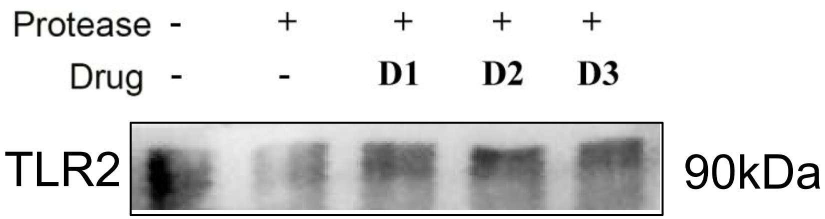

Group2

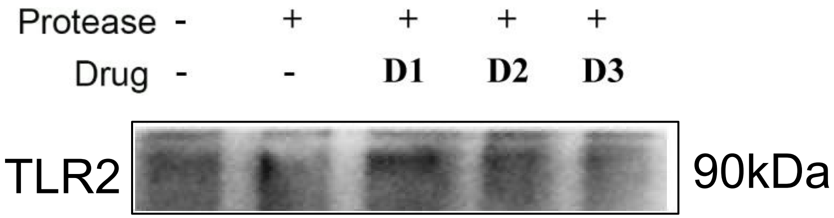

Group3

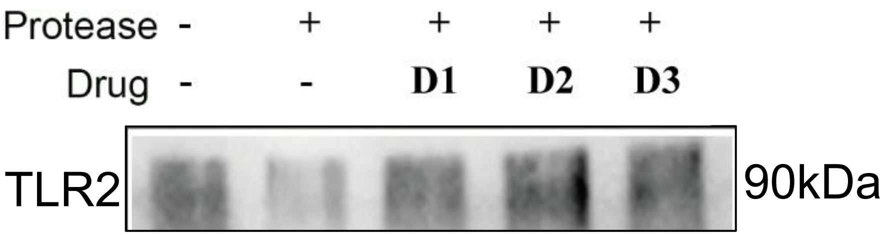

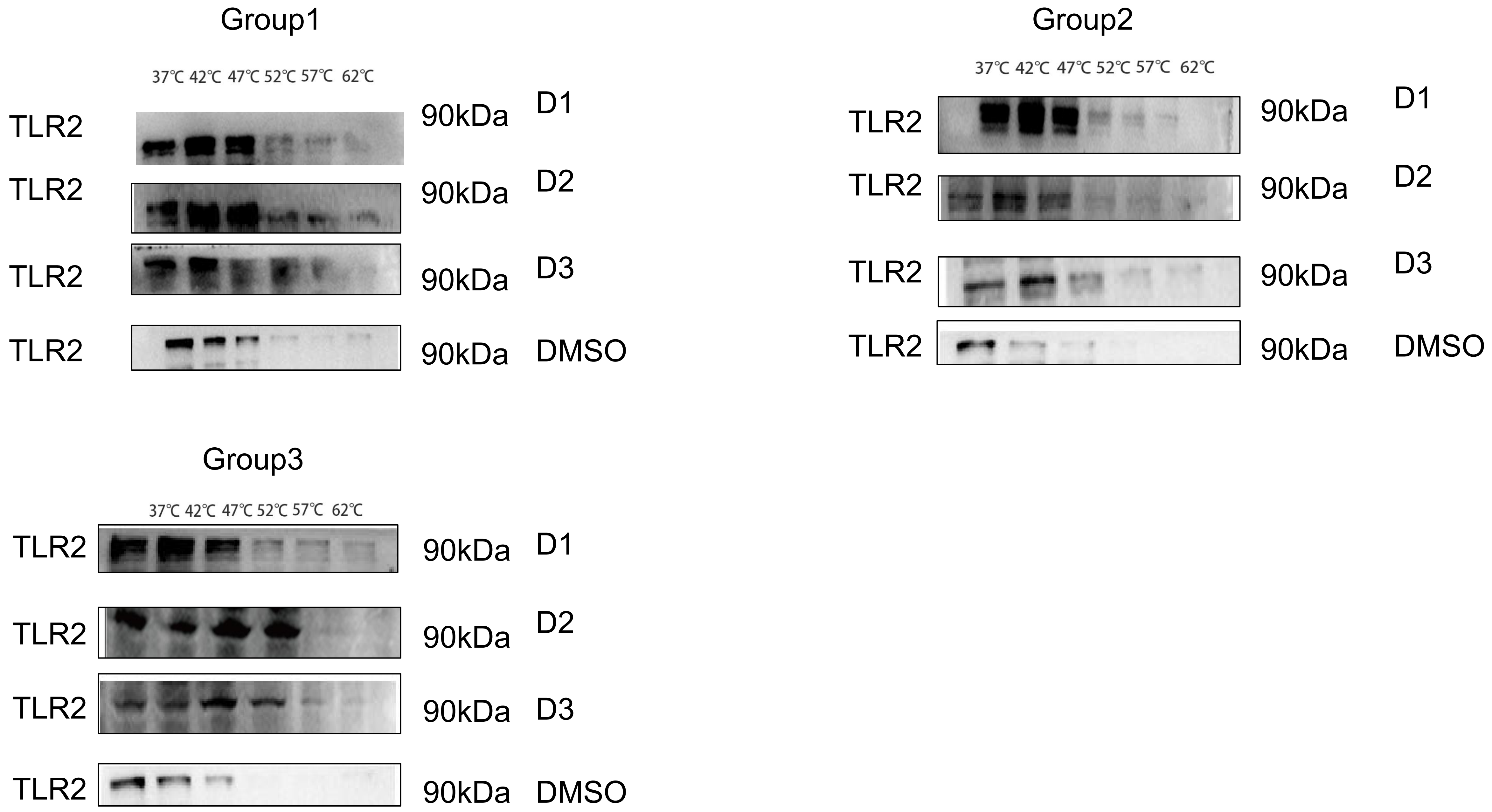
Group1
37℃ 42℃ 47℃ 52℃ 57℃ 62℃
TLR2 90kDa D1
TLR2 90kDa D2
TLR2 90kDa D3
TLR2 90kDa DMSO
Group2
37℃ 42℃ 47℃ 52℃ 57℃ 62℃
TLR2 90kDa D1
TLR2 90kDa D2
TLR2 90kDa D3
TLR2 90kDa DMSO
Group3
37℃ 42℃ 47℃ 52℃ 57℃ 62℃
TLR2 90kDa D1
TLR2 90kDa D2
TLR2 90kDa D3
TLR2 90kDa DMSO

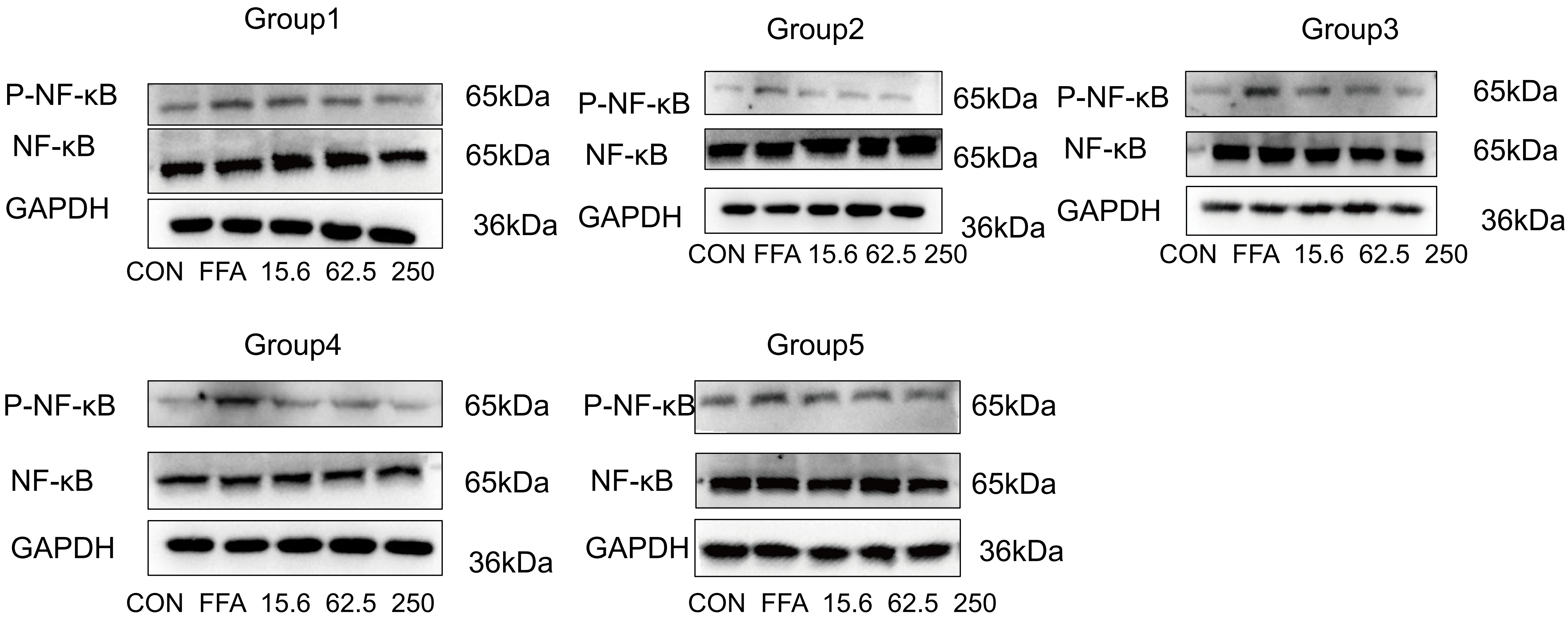
Group1
P-NF-κB
65kDa
NF-κB
65kDa
GAPDH
36kDa
CON FFA 15.6 62.5 250
Group2
P-NF-κB
65kDa
NF-κB
65kDa
GAPDH
36kDa
CON FFA 15.6 62.5 250
Group3
P-NF-κB
65kDa
NF-κB
65kDa
GAPDH
36kDa
CON FFA 15.6 62.5 250
Group4
P-NF-κB
65kDa
NF-κB
65kDa
GAPDH
36kDa
CON FFA 15.6 62.5 250
Group5
P-NF-κB
65kDa
NF-κB
65kDa
GAPDH
36kDa
CON FFA 15.6 62.5 250
